# Engaging Healthcare Staff and Stakeholders in Healthcare Simulation Modeling to Better Translate Research Into Health Impact: A Systematic Review

**DOI:** 10.3389/frhs.2021.644831

**Published:** 2021-11-23

**Authors:** Thea Zabell, Katrina M. Long, Debbie Scott, Judy Hope, Ian McLoughlin, Joanne Enticott

**Affiliations:** ^1^Monash Centre for Health Research and Implementation, Monash University, Clayton, VIC, Australia; ^2^School of Primary and Allied Health Care, Monash University, Frankston, VIC, Australia; ^3^Turning Point, Eastern Health and Eastern Health Clinical School, Monash University, Richmond, VIC, Australia; ^4^Monash Addiction Research Centre, Eastern Health Clinical School, Monash University, Frankston, VIC, Australia; ^5^Eastern Health Clinical School, Monash University, Box Hill, VIC, Australia; ^6^Mental Health Program, Eastern Health, Box Hill, VIC, Australia; ^7^Centre for Mental Health Education and Research, Delmont Private Hospital, Burwood, VIC, Australia; ^8^Department of Management, Faculty of Business & Economics, Monash University, Clayton, VIC, Australia; ^9^Department of Psychiatry, School of Clinical Sciences at Monash Health, Monash University, Clayton, VIC, Australia; ^10^Monash Partners Academic Health Science Centre, Clayton, VIC, Australia

**Keywords:** translation, simulation model, data driven healthcare organization (DDHA), data driven (DD), participatory research, healthcare improvement, stakeholder engagement

## Abstract

**Objective:** To identify processes to engage stakeholders in healthcare Simulation Modeling (SM), and the impacts of this engagement on model design, model implementation, and stakeholder participants. To investigate how engagement process may lead to specific impacts.

**Data Sources:** English-language articles on health SM engaging stakeholders in the MEDLINE, EMBASE, Scopus, Web of Science and Business Source Complete databases published from inception to February 2020.

**Study Design:** A systematic review of the literature based on a priori protocol and reported according to PRISMA guidelines.

**Extraction Methods:** Eligible articles were SM studies with a health outcome which engaged stakeholders in model design. Data were extracted using a data extraction form adapted to be specific for stakeholder engagement in SM studies. Data were analyzed using summary statistics, deductive and inductive content analysis, and narrative synthesis.

**Principal Findings:** Thirty-two articles met inclusion criteria. Processes used to engage stakeholders in healthcare SM are heterogenous and often based on intuition rather than clear methodological frameworks. These processes most commonly involve stakeholders across multiple SM stages *via* discussion/dialogue, interviews, workshops and meetings. Key reported impacts of stakeholder engagement included improved model quality/accuracy, implementation, and stakeholder decision-making. However, for all but four studies, these reports represented author perceptions rather than formal evaluations incorporating stakeholder perspectives. Possible process enablers of impact included the use of models as “boundary objects” and structured facilitation *via* storytelling to promote effective communication and mutual understanding between stakeholders and modelers.

**Conclusions:** There is a large gap in the current literature of formal evaluation of SM stakeholder engagement, and a lack of consensus about the processes required for effective SM stakeholder engagement. The adoption and clear reporting of structured engagement and process evaluation methodologies/frameworks are required to advance the field and produce evidence of impact.

## What is Known on This Topic

- Simulation modeling is an effective research methodology to address complex and “wicked” problems in healthcare and public health.
- Involving stakeholders in healthcare simulation modeling is assumed to produce better (and more relevant) models which are more readily accepted by problem owners and thereby more likely to be implemented, but there is limited evidence to guide choices of methods for engaging stakeholders to enhance the design and implementation of these models.

## What This Study Adds

- We document the large gap in the current literature of formal evaluation of SM stakeholder engagement, and a lack of consensus about the processes required for effective SM stakeholder engagement.
- Processes used to engage stakeholders in healthcare simulation modeling are heterogenous and often ill-defined in the literature, generally involving multiple stakeholder types across multiple simulation modeling stages.
- Possible process enablers of impact are the use of models as “boundary objects” and structured facilitation *via* storytelling for non-technical communication of model logic. These enablers may work by providing a common language and mutual understanding between stakeholders and modelers.
- Adoption and clear reporting of structured engagement and process evaluation methodologies/frameworks are required to advance the field.

## Introduction

Healthcare decision-makers are facing increasingly “wicked” problems, which have both a technical (complex and uncertain symptoms and solutions) and social (divergent stakeholder perspectives) dimensions (1, 2). Confronting the technical dimension requires research methods which can account for scientific complexity and uncertainty whilst addressing the social dimension requires stakeholders to be engaged in the research process in order to produce solutions with real-world utility (3, 4).

Simulation Modeling (SM) is an established but historically under-utilized methodology in healthcare (5). SM creates a virtual environment which captures dynamic, interdependent and emergent system behaviors in formal models or mathematical representations (5, 6). These models can be used to “advance the understanding of the system or process, communicate findings, and inform management and policy design” (6). SM comprises three methods—system dynamics, discrete event simulation, and agent-based modeling—which Marshall et al. claim are “well suited to healthcare delivery problems” (6).

SM has increasingly been combined with approaches intended to engage stakeholders in the modeling process. Engaging stakeholders, traditionally managers and clinicians in the relevant healthcare field, has been claimed to yield both more technically and socially robust simulation solutions (7) and improve on the poor model implementation that has plagued SM for many years (5, 7–11). Barreteau et al. outline three expected benefits of combining a participatory process with SM: (1) to upgrade the quality of a simulation model, (2) to improve the suitability of the simulation model's use (implementation), and (3) to support participation itself, and account for different perspectives (function of models within participatory process) (12). Despite these expected benefits, SM stakeholder engagement research and practice in healthcare lags behind other sectors (e.g., defense and commerce) (13).

Knowing how best to involve themselves or others in research is a challenge for clinicians, decision-makers and stakeholders in healthcare, as well as for the researchers. Yet involving frontline clinicians, decision-makers and other relevant stakeholders in research that aims to promote a change in practice is key to translating research “off the shelf and into practice” (14). Several authors have identified barriers to successful stakeholder engagement unique to SM research. Jahangirian et al. determined the primary causal factor of poor engagement as the “communication gap between simulation and stakeholder groups” as simulation modelers may have particularly technical backgrounds (9). Brailsford et al., drawing on their experiences within the UK, discuss commonly encountered barriers, including cultural differences and ethical hurdles (8). Whilst an understanding of barriers to engagement is important, guiding decisions in practice about how to effectively engage different stakeholders in designing healthcare simulation models requires further understanding of who should be engaged, when this engagement should occur, and how this engagement should be done to generate the intended impacts. Several simulation studies in healthcare have provided descriptions, reflections or evaluations of their stakeholder engagement process (15, 16), however, there is no coherent body of literature in this area.

The aim of this review is to systematically synthesize the evidence on how far and by what means stakeholder engagement in SM results in outcomes with more practical utility and prospect of successful implementation. A key objective is to identify the processes used to meaningfully engage stakeholders in SM research and to analyze the impacts of this engagement in enhancing the design and implementation of healthcare simulation models. In order to accomplish this, we analyzed the extent to which these intended purposes or expected benefits of SM have materialized in applications to healthcare problems, and we identify the contribution of engagement processes to facilitating this.

## Methods

### Study Design

This systematic review is reported according to the PRISMA statement (17) and used an a priori established protocol (Supplementary Material).

### Eligibility Criteria and Search Strategy

Eligible articles were original studies that (1) used dynamic SM (intervention), (2) reported a health-related primary outcome (context), (3) engaged stakeholders during the *model design* stage (population), and (4) reported stakeholder engagement impact (outcome). The search was not limited to a specific study design and did not include a comparator. English-language articles were searched in MEDLINE, EMBASE, Scopus, Web of Science and Business Source Complete databases published from inception to February 2020. Common keywords included: simulation, system dynamics, discrete event or agent based; health care, healthcare, hospital, primary care, public health, health policy or health service; group model building, stakeholder, client, customer, implementation, focus group, interview, steering group, advisory board, advisory committee, co-design, co-production; and participatory simulation or participatory model. Full details are in Appendix A including the title and abstract screening criteria and the full text inclusion criteria.

### Study Selection, Data Extraction and Analysis

Title and abstract, and then full-text screening (see Appendix A for full details) were conducted by TZ (all studies) and KL (25% of studies), with disagreement resolved by discussion. Data were extracted using a data extraction form adapted from Concannon et al. (18) and located in Appendix A.

**Figure 1:**
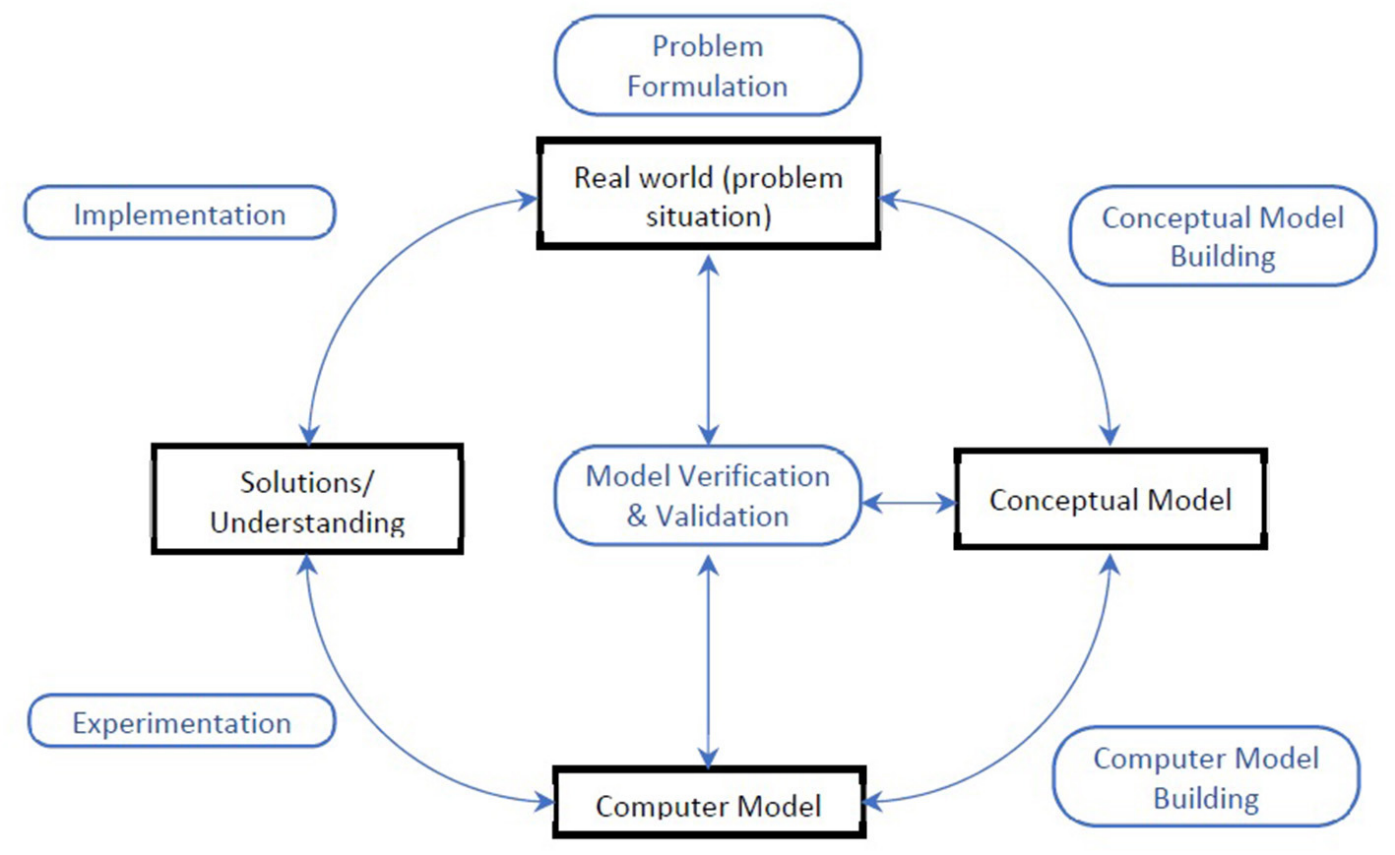
Generic stages in SM.

Data were analyzed using summary statistics, deductive and inductive content analysis, (19) as well as a narrative synthesis approach (20). Summary statistics were used to analyze study characteristics. Content analysis was applied to synthesize qualitative data describing the participatory process and intended or reported impacts of this process. We used matrices to explore the overlap between process characteristics and intended or reported impacts, in order to map how the nature of the process may link to impacts. To obtain a richer understanding of the participatory processes, a narrative synthesis approach was used to analyze the role of stakeholder engagement activities within the SM process. Topic areas were categorized according to Mielczarek and Uziałko-Mydlikowska (21), and stakeholder types were categorized according to an adaptation of the 7P's framework, with purchasers and payers combined into a single category (14). The generic stages of SM lifecycles (Figure 1) were used to represent modeling stages– problem formulation, conceptual modeling, computer modeling, model verification and validation, experimentation and implementation (22). Other stages which engaged stakeholders that didn't fit into the generic stages were inductively coded. The intended and reported benefits of the participatory process were coded within a framework adapted from Barreteau et al., comprising three broad types of benefits for (1) the design of the model, (2) the implementation of the model, and (3) the stakeholder participants (12). Within this framework, inductive content analysis was used to identify and quantify the sub-groups of benefits.

The evidence synthesis concentrated on authors' reporting of the participatory process in SM studies, which meant that outcome measures from the studies were not included. Therefore, no formal assessment of risk of bias was necessary either in individual studies or across studies (23).

## Results

**Figure 2:**
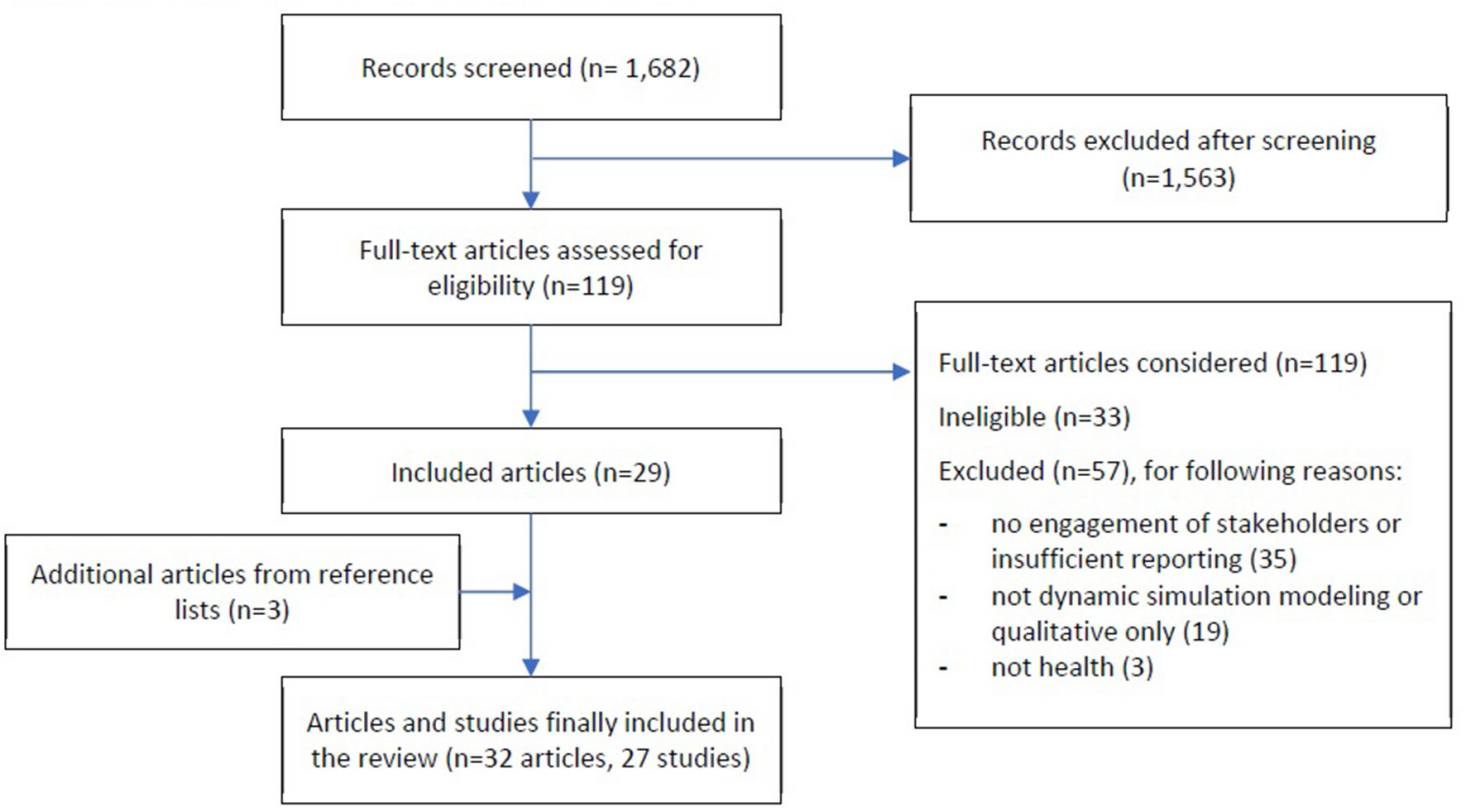
PRISMA flow diagram of study selection.

The search yielded 1,682 titles and abstracts for initial screening, with 119 articles included for full-text screening. Of full-text articles screened, 29 met the eligibility criteria, with a further three identified and included from included articles reference lists (see Figure 2 PRISMA diagram). The final 32 articles reported on 27 studies (see Table 1 for a summary of included studies).

**Table 1:** Summary of studies included in the systematic review (*n* = 27).

**References and country**	**Model**			**Stakeholders**		**Stakeholder engagement**		**Impacts**
	**Topic area**	**Type**	**Health-related outcomes**	**Type and number**	**Recruitment method**	**Stages**	**Modes and analysis**	
Atkinson et al. (24); Freebairn et al. (15, 25) Australia	Epidemiology, health promotion and disease prevention	Agent-Based Modeling	Alcohol-related harms, violence, ED presentations, and hospitalisations	Policymakers, PIs, Providers. N = 10–15 (planned)	Purposive sampling	Problem formulation, Conceptual MB, Model Verification, Experimentation/ Implementation, Parameterisation, Participant recruitment	Discussion/ dialogue, Meetings, Workshop Structured Direct model interaction	**Model design:** Model quality, Data identification, Multiple perspectives **Participants:** Productive discussion, Learning, Problem-solving **Implementation:** Model acceptance, Implementation
Baldwin et al. (26, 27) UK	Health and care systems operation	Discrete event simulation	Cost-effectiveness of liver transplant patients wait list prioritization models	Providers, Purchasers/ Payers. N = ns	Purposive sampling	Problem formulation, Conceptual MB, Model Verification, Experimentation/ Implementation	Discussion/ dialogue ns Direct model interaction	**Model design:** Problem relevance, Multiple perspectives **Participants:** Productive discussion
Barbrook-Johnson et al. (28) UK, USA, EU	Epidemiology, Health Promotion and Disease Prevention	Agent-Based Modeling	Influenza infections, vaccination-seeking, individual adoption of other protective behaviors	Policymakers, PIs, Providers, Purchasers/ Payers. N = 48	Purposive sampling	Conceptual MB, Model Verification, Experimentation/ Implementation	Questionnaire, Workshop ns Thematic analysis, Direct model interaction	
Bell et al. (29) UK	Health and care systems operation	Hybrid (System Dynamics, Discrete Event Simulation)	ED presentations, unplanned hospital admissions, hospital readmissions, bed occupancy	Providers, Purchasers/ Payers. N = ns	Actor inheritance	Problem formulation, Conceptual MB, Model Verification	Interviews ns Direct model interaction	**Model design:** Model quality
Bowers et al. (30) UK	Health and care systems operation	Discrete Event Simulation	ED Patient wait time	Providers. N = ns	Accessed through collaborative institution, As part of a larger project/ initiative	Conceptual MB, Model Verification, Experimentation/ Implementation	Discussion/ dialogue Unstructured Direct model interaction	**Model design:** Model quality **Implementation:** Model acceptance, Implementation
de Andrade et al. (31) Brazil	Health and Care Systems Operation	System Dynamics	ST-segment elevation myocardial infarction patient ejection fraction, length of stay	Providers. N = 6	Convenience sampling, Accessed through collaborative institution	Problem formulation, Conceptual MB	Interview Structured ns	**Model design:** Multiple perspectives
Freebairn et al. (15, 25, 32, 33) Australia	Epidemiology, health promotion and disease prevention	Hybrid (Discrete event simulation, system dynamics, agent-based modeling)	Gestational diabetes incidence and later-life type 2 diabetes incidence, offspring gestational diabetes and type 2 diabetes incidence	Policymakers, Providers, Purchasers/ Payers N = 10–15 (planned)	Purposive sampling	Problem formulation, Conceptual MB, Model Verification, Experimentation/ Implementation, Parameterisation, Process Evaluation	Discussion/ dialogue, Interview, Meetings, Workshop Structured Thematic analysis, Direct model interaction	**Model design:** Problem relevance, Model quality, Data identification, Multiple perspectives **Participants:** Productive discussion, Learning, Problem-solving **Implementation:** Model acceptance, Implementation
Giesen et al. (34) Netherlands	Health and care systems operation	Agent-Based Modeling	Provision of youth health care to difficult cases, wait list size, patient withdrawal from wait list, patient wait time, provider utilization	Providers. N = 4	ns	Conceptual MB, Model Verification	Discussion/ dialogue, Interviews ns Direct model interaction	**Model design:** Model quality
Glasgow et al. (35) UK	Extreme Events Planning Health and Care Systems Operation	Discrete Event Simulation	Exhaustion of red blood cell inventory, adherence to blood transfusion guidelines	PIs, Providers. N = ns	Accessed through collaborative institution	Conceptual MB, Model Verification, Parameterisation	Discussion/ dialogue, Questionnaire Unstructured ns	**Model design:** Model quality **Participants:** Problem-solving
Hassmiller Lich et al. (36) USA	Epidemiology, health promotion and disease prevention	System dynamics	Prevalence of youth with managed serious emotional disturbance	Patients/ Public, PIs, Providers. N = 103	Public advertising	Problem formulation, Conceptual MB, Experimentation/ Implementation	Webinar, Workshop Structured Value coding, Thematic analysis	**Participants:** Productive discussion **Implementation:** Refined terminology
Homa et al. (37) USA	Epidemiology, Health Promotion and Disease Prevention Health and Care Systems Design	Agent-Based Modeling	Average patient health (all patients; patients with chronic illnesses), clinician visits (total; due to poor health), primary care visits resulting in specialist referrals	Patients/ Public, Providers. N = 15	Purposive sampling	Problem formulation, Conceptual MB, Model Verification, Experimentation/ Implementation	Focus groups, Interviews, Workshop Structured Direct model interaction	**Model design:** Multiple perspectives
Hung et al. (38) Canada	Health and Care Systems Operation	Discrete Event Simulation	Pediatric ED patient wait time, length of stay	Providers. N = ns	As part of a larger project/ initiative	Conceptual MB	Interviews Unstructured ns	
Johnson et al. (39) UK	Health and care systems operation	Discrete event simulation	Total cost of treatment pathways for sepsis, pneumonia, chemotherapy	Providers, Purchasers/ Payers. N = ns	Purposive sampling, As part of a larger project/ initiative	Problem formulation, Conceptual MB, Model Verification, Parameterisation	Meetings ns Direct model interaction	**Model design:** Model quality, Data identification
Lane et al. (16) UK	Health and care systems operation	System dynamics	ED Patient wait time, elective cancellations, hospital occupancy; ED clinician utilization	Providers. N = 14	Accessed through collaborative institution	Conceptual MB, Model Verification, Experimentation/ Implementation, Parameterisation	Discussion/ dialogue, Meetings Unstructured Direct model interaction	**Model design:** Model quality **Participants:** Learning, Problem-solving **Implementation:** Model acceptance
Lattimer et al. (40) UK	Health and care systems operation	System dynamics	ED throughput, hospital bed occupancy	Providers. N = 30 (interviews) N = ns (discussion)	Accessed through collaborative institution	Conceptual MB, Computer MB, Experimentation/ Implementation, Parameterisation	Discussion/ dialogue, Interviews Unstructured Direct model interaction	**Model design:** Model quality **Participants:** Productive discussion
Leskovar et al. (41) Slovenia	Health and care systems operation	Discrete Event Simulation	Hospital administrative staff utilization	Ns. N = ns	ns	Problem formulation, Conceptual MB	Interviews ns ns	
Lote et al. (42) USA	Health and care systems operation	Discrete Event Simulation	Medical laboratory staff utilization across departments	Providers. N = ns	ns	Conceptual MB, Model Verification	Discussion/ dialogue ns Direct model interaction	
Mackay et al. (43) Australia	Health and care systems operation	Hybrid (System Dynamics, Discrete Event Simulation, Agent-Based Modeling)	ED patient wait time, hospital bed occupancy	Ns. N = ns	ns	Conceptual MB, Model Verification, Experimentation/ Implementation, Parameterisation	ns ns Direct model interaction	**Implementation:** Implementation
Matchar et al. (44) Singapore	Health and care systems operation	System dynamics	Proportion of population with complex condition, cost per person, patient satisfaction, doctor-patient relationship	Patients/ Public, Policymakers, PIs, Providers, Purchasers/ Payers. N = 50	ns	Problem formulation, Conceptual MB	Discussion/ dialogue, Workshop Structured Direct model interaction	**Model design:** Multiple perspectives
Mitchell et al., (45) USA	Epidemiology, Health promotion and disease prevention	System dynamics	Rates of adolescent screening for alcohol, tobacco and substance abuse problems, positive screenings, brief interventions	Providers. N = ns	As part of a larger project/ initiative	Problem formulation, Conceptual MB, Model Verification, Experimentation/ Implementation	Interviews, Meetings, Webinar ns ns	
Murphy et al. (46) Jamaica	Health and care systems operation	System dynamics	Gap between available and required registered nurses	Policymakers, PIs, Purchasers/ Payers. N = ns	As part of a larger project/ initiative	Problem formulation, Conceptual MB, Model Verification, Experimentation/ Implementation, Parameterisation	Discussion/ dialogue ns ns	
Roberts et al. (47); Freebairn et al. (15, 25) Australia	Epidemiology, health promotion and disease prevention	System dynamics	Prevalence of overweight and obese children	Policymakers, PIs, Providers, Purchasers/ Payers. N = 44	Accessed through collaborative institution	Problem formulation, Conceptual MB, Model Verification, Experimentation/ Implementation, Parameterisation	Workshop ns Direct model interaction	**Model design:** Multiple perspectives **Participants:** Productive discussion, Learning, Problem-solving **Implementation:** Model acceptance, Implementation
Rosmulder et al. (48) Netherlands	Health and care systems operation	Discrete event simulation	ED Patient length of stay, quality of care	PIs, Providers. N = 6	Accessed through collaborative institution	Problem formulation, Conceptual MB, Model Verification, Experimentation/ Implementation	Discussion/ dialogue, Meetings, Public Display, Workshop Unstructured Direct model interaction	**Model design:** Multiple perspectives **Participants:** Problem-solving **Implementation:** Implementation
Rwashana et al. (49); Semwanga et al. (50) Uganda	Epidemiology, health promotion and disease prevention	System dynamics	Neonatal mortality	Patients/ Public, Policymakers, PIs, Providers. N = 345	Random sampling, Convenience sampling, Purposive sample	Problem formulation, Conceptual MB, Computer MB, Model Verification, Experimentation/ Implementation, Process Evaluation	Interview, Workshop ns Thematic analysis, Direct model interaction	**Model design:** Problem relevance **Participants:** Productive discussion, Problem-solving
Uebelherr et al. (51) USA	Epidemiology, health promotion and disease prevention	Agent-Based Modeling	Cooling center coverage during extreme heat	Policymakers, Providers. N = ns	Snowball sampling	Problem formulation, Conceptual MB	Interviews ns ns	**Model design:** Multiple perspectives **Participants:** Problem-solving
Uriarte et al. (52) Sweden	Health and care systems operation	Discrete event simulation	ED patient wait time, length of stay	Ns. N = ns	Accessed through collaborative institution	Problem formulation, Conceptual MB, Model Verification, Parameterisation	Discussion/ dialogue, Meetings ns ns	**Participants:** Problem-solving **Implementation:** Implementation
Zimmerman et al. (53) USA	Health and care systems operation	System Dynamics	Evidence-based psychotherapy initiation and completion in veterans	Patients/ Public, Providers. N = ns	Accessed through collaborative institution	Problem formulation, Conceptual MB, Computer MB, Model Verification, Experimentation/ Implementation, Parameterisation	Meetings Structured Qualitative formative evaluation, Direct model interaction	**Model design:** Problem relevance, Data identification **Participants:** Productive discussion, Learning, Problem-solving **Implementation:** Implementation

*UK, United Kingdom; USA, United States of America; EU, European Union; ED, Emergency Department; PIs, Principal Investigators; MB, Model Building; ns, not specified*.

### Study Characteristics

**Figure 3:**
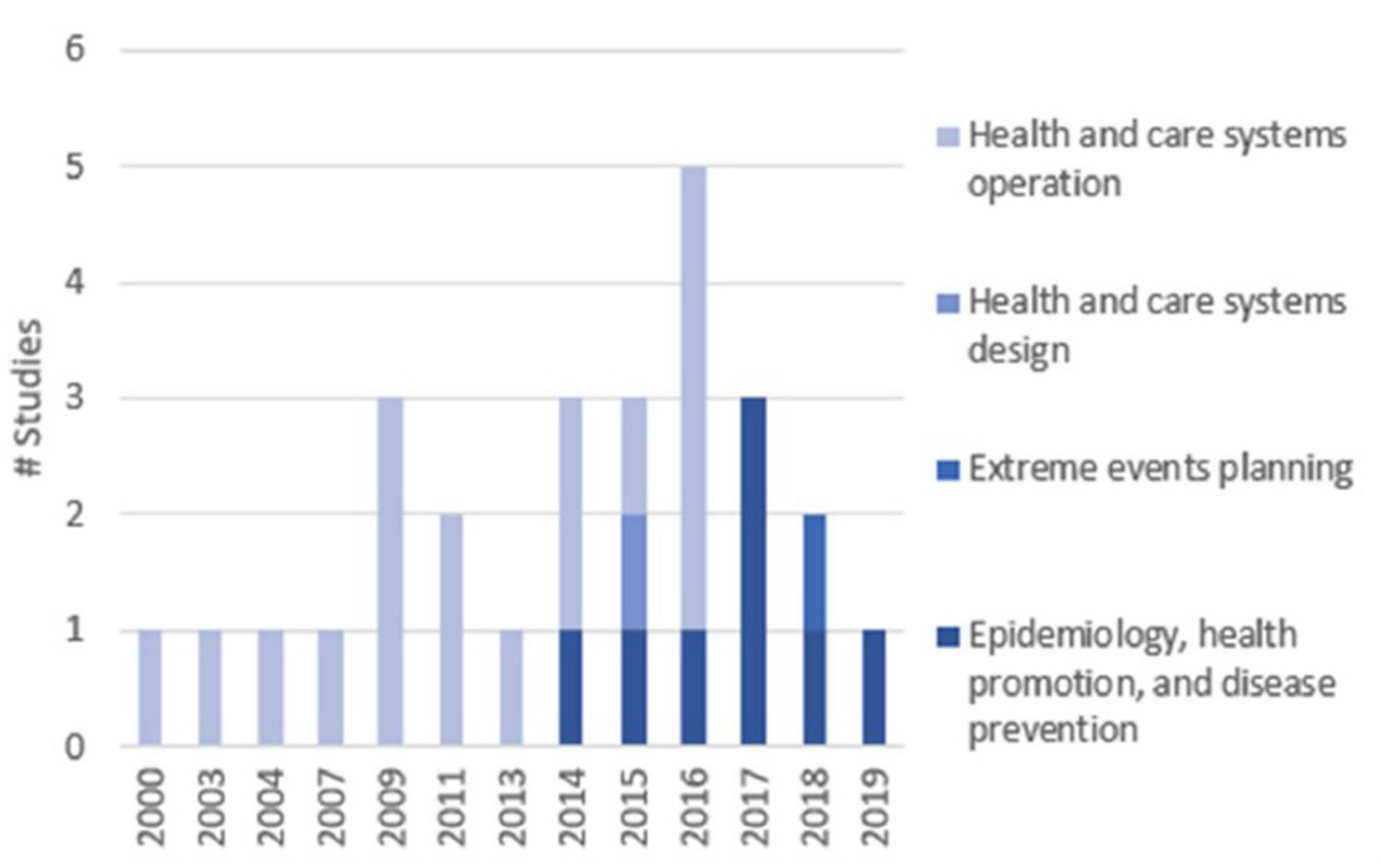
Evolution of study topic areas over time.

Of the 27 studies included, the majority were conducted in the UK (*n* = 8, 30%), US (*n* = 7, 26%) and Australia (*n* = 4, 15%). The most common topic areas were *Health and Care Systems Operation* (56%) and *Epidemiology, Health Promotion and Disease Prevention* (41%), with few studies in *Health and Care Systems Design* (3%) and *Extreme Events Planning* (3%). None of the included studies addressed the topic of *Medical Decision Making*. Years of publication ranged from 2000 to 2019, with 78% published since 2014 (*n* = 21). There was a change in trend in study topic areas over time: initially dominated by *Health and Care Systems Operation*, and in recent years by *Epidemiology, Health Promotion and Disease Prevention* (Figure 3).

### Stakeholder Participants

**Figure 4:**
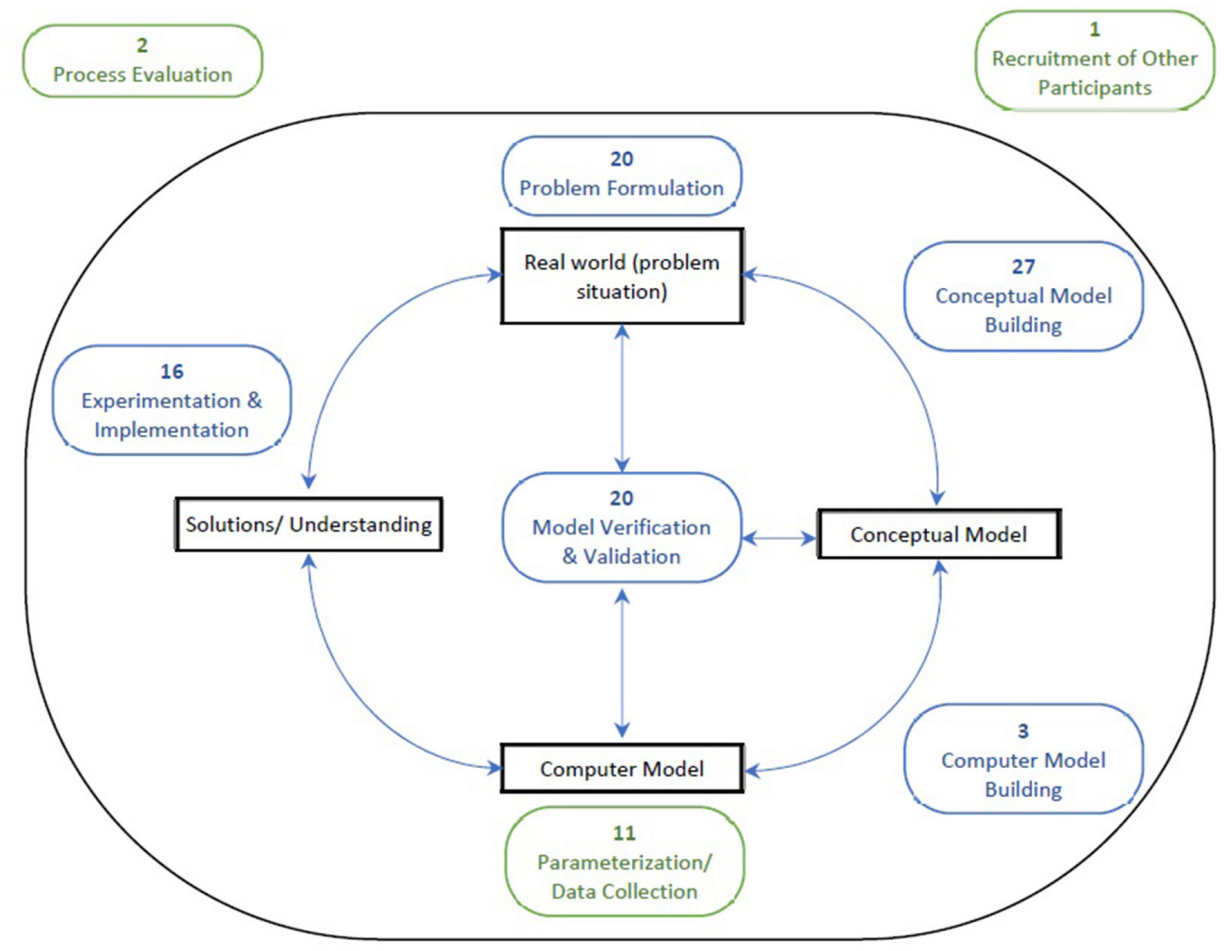
Number of studies reporting stakeholder engagement during generic (blue) and openly coded (green) stages of SM. Common stages of engagement aside from conceptual model building were problem formulation (74%). model verification & validation (74%) and experimentation & implementation (59%). It was relatively uncommon for studies to engage stakeholders during the computer model building stage (11%). however, collecting data or parameters through the engagement of stakeholders was used by almost half the studies as a means of quantifying the computer model (41%). Only two studies (7%) engaged stakeholders in evaluating the participatory process in the simulation study.

The number of studies reporting stakeholder engagement during different stages of SM are shown in Figure 4. The type and number of stakeholders involved in the participatory process varied widely between studies. The most frequently-engaged stakeholders were Providers (*n* = 23), with less engagement with Policy-makers (*n* = 7), Purchasers/Payers (*n* = 9) and Patients/Public (*n* = 6). Each study, on average, engaged two different types of stakeholder participants. Further details on the numbers and types of stakeholders are in Appendix B. Recruitment methods for stakeholders are also in Appendix B.

### The Participatory Process: Stakeholder Engagement Stages, Modes and Activities

The stages during which stakeholders were engaged are depicted graphically in Figure 4. All but one study engaged stakeholders in more than one stage of the SM process. In nine studies (33%), stakeholders were engaged in all the generic stages (from the beginning to the end of the SM lifecycle—excluding computer model building). There were four primary modes employed by studies in the engagement of stakeholders: discussion/dialogue (*n* = 13, 48%), interviews (*n* = 11, 41%), workshops (*n* = 8, 30%), and meetings (*n* = 7, 26%), with some variation across SM stages. Specifically, interviews were most common earlier in the SM process while workshops were commonly used in the mid and late stages of the process. Workshops, discussion/dialogue and meetings generally involved direct model interaction, where the model acted as a communication vehicle (*n* = 18, 67%), allowing stakeholders to physically manipulate and “play” with the model design. Some studies provided descriptions about how they facilitated this input, which ranged from structured and active methods where stakeholders were asked specific questions (31) or engaged in purposeful storytelling exercises (32), to unstructured and passive methods where stakeholders provided feedback about or annotated an existing model (30, 40). More structured methods of facilitation were used in early stages when studies were engaging stakeholders in designing the model from scratch (24, 31, 32, 36, 37, 44, 53), and more passive methods were used when stakeholders were engaged at a later stage and a draft model had already been designed (16, 30, 35, 38, 40, 48). Further details about the Modes of Engagement & Facilitation are found in Appendix C.

### Assessing the Impacts of Stakeholder Engagement

There were four types of impacts reported from stakeholder engagement on model design: (1) increased relevance of the problem addressed (*n* = 4, 14%), (2) better quality/accuracy of the model for its purpose (*n* = 9, 33%), (3) improved identification or access to better data (*n* = 4, 15%), and (4) expertise from a range of perspectives (*n* = 9, 31%). Across the SM stages, increased relevance of the problem was most reported during problem formulation, while better quality/accuracy of the model was most reported during the conceptual modeling phase.

There were three types of impacts reported from the participatory process on the stakeholder participants: (1) productive discussion or shared understanding of the problem (*n* = 8, 28%), (2) “learning” (*n* = 5, 17%), and (3) better problem solving or decision-making (*n* = 10, 35%). The fact that models require perspectives and assumptions to be made explicit in a graphical representation which imitates the real system meant that several studies found that interacting with the model created productive discussion between stakeholders, particularly those from different disciplines (24–26, 49). There were also three types of impacts reported from stakeholder engagement on model implementation: (1) refined use of terminology (*n* = 1, 3%) (36), (2) greater acceptance or ownership of the model (*n* = 5, 17%), and (3) improved implementation or suitable use of the model (*n* = 8, 27%).

In the overlap between the participatory process and impacts, studies involving direct model interaction were more likely to report benefits for stakeholder ownership of the model (56 vs 11%) and productive discussion & shared understanding (61 vs. 22%), vs. studies without direct model interaction. Read more about the impacts of participatory process on model design, implementation and participants in Appendix D.

### Specific Processes Used to Engage Stakeholders in Simulation Modeling

Some studies provided specific details about the process used to combine stakeholder engagement and SM and how to do this well (26, 29, 32, 33, 49). This can provide practitioners and decision-makers, as well as researchers, with useful guides for engaging in such processes. These processes included: the Collaborative Hybridization Process (29), an adapted dynamic synthesis methodology (49), and the Modeling Approach that is Participatory Iterative for Understanding (MAPIU) (26). Descriptions and examples of these processes are outlined in Appendix E.

## Discussion

We have systematically reviewed the ways in which studies using SM have engaged stakeholders through participatory processes. We reviewed these participatory processes on their reported abilities to improve the design and use of models in healthcare as well as produce desired impacts on stakeholder participants. The reported processes used to engage stakeholders in healthcare SM were heterogenous, but there were common characteristics in terms of the stages, modes and activities through which engagement is facilitated. Studies mostly commonly involved provider stakeholders, across multiple SM stages, using discussion/dialogue, interviews, workshops and meetings as key modes of engagement. In addition to conceptual modeling, many studies engaged stakeholders in the adjacent stages of problem formulation and model verification and validation, as well as during the later stages of experimentation and implementation. Interviews were mostly used earlier in the SM process while workshops were mostly used in the mid and late stages of the process. Key reported impacts of stakeholder engagement included improved model quality/accuracy, implementation, and stakeholder decision-making.

### The Link Between Stakeholder Engagement Process and Impact

The communication gap between stakeholders and modelers has been identified as a primary causal factor driving consistently poor rates of model implementation in healthcare SM (9, 54). This review has identified two possible mechanisms by which engagement processes, *via* improved model design and stakeholder impacts, may lead to improved model implementation.

Firstly, we found that direct interaction between stakeholders and the model seemed to influence interpersonal communication (between stakeholders themselves and between stakeholders and modelers), leading to stakeholder impacts of more productive discussion and shared understanding, and implementation impacts of greater ownership and acceptance of the model. This is likely because the model operates as a “boundary object” (55), a visual “multi-interpretable, consistent transparent, and verifiable representation of reality” (56). To effectively use models in this way, Rose et al. recommend: using specific conventions to describe model components and interactions to create a common language; using an early simple model to teach stakeholders the model concepts; and allowing hands-on stakeholder exposure to the model user interface (57). As such, using models as “boundary objects” may provide structures around which to base effective communication, providing visual aids for stakeholders to view the whole problem system and better identify areas for solutions.

More structured methods of stakeholder engagement, i.e., the use of specific questions (31) or purposeful storytelling exercises (32), were also associated with improved quality of models for their purpose and helped to incorporate diverse perspectives and expertise into the model design. Freebairn et al. discussed a particularly effective example of using “storytelling” to facilitate communication of the model structure through clinical case histories of individuals–a thought process familiar to clinical stakeholders (32) During storytelling the modelers are better able to use language that the stakeholders can understand and relate to. Also, having the stories allows stakeholders to give the modelers an increased understanding of the complexity of “wicked” problems and complexities associated with populations affected by these problems. The use of storytelling can exemplify the individual trajectories of agents, communicating the ability of the model to capture the evolution of agents over time. During the process evaluation, participants reported that while the sophisticated and highly technical nature of the model remained a barrier in communicating easy to understand policy messages, the use of storytelling to compliment the model outputs was a “particularly valuable tool” to improve mutual understanding of the model (32). That greater understanding contributes to improving the modeling, confidence in the model and ownership. This example suggests that storytelling may provide a useful addition to the “boundary object” approach, allowing communication of highly technical model elements in a more easily understood way. It's also likely to contribute to successful design, confidence, model ownership and future implementation as stories seem to get the message across people from different disciplines.

### Reporting and Evaluation of Stakeholder Engagement in SM in the Literature

Many of the methodological issues faced by this review were due to a lack of standardized or detailed reporting of stakeholder engagement, and insufficient reporting was one of the primary reasons for article exclusion. This lack of reporting comprised the details of the process itself and adequate evaluations of the engagement process. Only five studies provided specific details about the stakeholder engagement process (26, 29, 32, 33, 49), and a mere four studies reported on a process evaluation from the stakeholder perspective (15, 30, 49, 53). For the remaining studies, it was difficult to distinguish between intended and reported impacts that were observed or realized during the process as the reporting was based solely on the authors' reflections.

A recent framework from the environmental model building field provides a possible solution to the lack of standardized reporting of stakeholder engagement that may be equally applicable to healthcare SM. Gray et al. propose a four-dimensional reporting framework (4P) which includes: (1) the Purpose for using a participatory SM approach (i.e., intended impacts), (2) the Process by which stakeholders were involved in model building or evaluation; (3) the Partnerships that were defined and participants that were chosen; and (4) the Products that resulted from these efforts (i.e., actual impacts) (58). A detailed breakdown of each of these dimensions is provided by the authors in addition to 4 exemplar case studies. This could be supported by the adaptation of one of the engagement processes identified in our review. The MAPIU is easily generalizable and provides several frameworks for designing a participatory SM process, including a classification system for stakeholders, and frameworks for how different stakeholder contributions fit into the MAPIU process and what types of communication should be considered (26). Guidance on process evaluation is sparser, with Esmail et al.'s systematic review identifying only two studies reporting quantitative results, with most formal evaluations relying on qualitative, self-reported retrospective accounts of the engagement experience (59). Future research should focus on the development and validation of measures and methods for rigorous evaluation of engagement in healthcare SM which should be an *a priori* embedded component of the research design.

A limitation of this review is the timeframe, which included studies published until March 2020.The crisis and transformation occurring in health care since February 2020 due to the COVID-19 pandemic is deliberately not captured here. The COVID-19 pandemic has resulted in rapid changes inside health systems including changes in direct care procedures and the adoption of remote care through new technologies, with a corresponding global burden of high health care worker stress. Therefore, health care stakeholder involvement for SM during COVID-19 would involve different, crisis driven approaches, and is the subject of a separate subsequent project.

## Conclusion

This review explored the process by which studies engaged stakeholders in healthcare SM and the impacts of the engagement process on model design, model implementation and stakeholders. We found that engagement of stakeholders in the SM process was common during multiple stages, involved informal discussion as well as more formal one-to-one interviews or group workshops, and was facilitated by a range of more or less structured activities for model building, with structured activities associated with improved model quality and ability to capture diverse perspectives and expertise. Key enablers reported by authors and stakeholder participants were the use of the evolving model as a “boundary object” to facilitate communication and storytelling to communicate the model logic, complexities and interactions in a non-technical way. We suggest the adoption and clear reporting of structured engagement and process evaluation methodologies/frameworks to enable high-quality stakeholder involvement, improve SM confidence and ownership by healthcare staff and decision-makers, and ultimately lead to implementation of optimal interventions identified by SM that contribute toward better health care systems.

## Data Availability Statement

The original contributions presented in the study are included in the article/Supplementary Material, further inquiries can be directed to the corresponding author.

## Author Contributions

TZ, KL, IM, and JE devised the research and developed the research questions in collaboration with DS and JH. Search strategy was devised by TZ with assistance by KL and JE. Articles were screened by TZ and KL and fulltext extraction performed by TZ with technical guidance and assistance by KL and JE. Manuscript was drafted by TZ. All authors provided inputs into subsequent revisions.

## Conflict of Interest

The authors declare that the research was conducted in the absence of any commercial or financial relationships that could be construed as a potential conflict of interest.

## Publisher's Note

All claims expressed in this article are solely those of the authors and do not necessarily represent those of their affiliated organizations, or those of the publisher, the editors and the reviewers. Any product that may be evaluated in this article, or claim that may be made by its manufacturer, is not guaranteed or endorsed by the publisher.
