# Supplementary material for: Engaging Healthcare Staff and Stakeholders in Healthcare Simulation Modeling to Better Translate Research Into Health Impact: A Systematic Review
Source: Front Health Serv. 2021 Nov 23;1:644831. doi: 10.3389/frhs.2021.644831 (PMC10012644; doi:10.3389/frhs.2021.644831)
Supplement: Supplementary file 2 [file Data_Sheet_2.docx]

**Appendix A: Additional details on the Methods**

**Eligibility Criteria for studies in this review**

Original studies from all years, which had a published full-text in English language, were included. Other types of literature, including literature reviews, theoretical protocols, comments, newsletters, opinions, letters and editorials, were excluded. Studies which were only reported as abstracts or poster abstracts were also excluded. A non-PICOS framework was used in defining the inclusion and exclusion criteria in order to make it more appropriate to the SM literature.

Eligibility was contingent three criteria: (1) dynamic SM was used, (i.e. system dynamics, discrete-event simulation, agent-based modeling, or hybrid modeling including at least one of these), (2) public health or healthcare was the focus of the simulation model, including a health-related primary output, (3) stakeholders were engaged during the *model design* stage, which meant they provided input which was incorporated into, or led to changes in, model variables or structure, 4) impacts of stakeholder engagement were reported. The lack of health-related output for the health criteria led to exclusion, for example, Badland et al. was excluded because “walkability” of a neighborhood was the output optimized, rather than the health-related consequences of increasing the walkability^1^. Participation in surveys was considered to be stakeholder engagement if this data was collected for the main purpose of using it to inform model design. Papers were excluded if they provided insufficient detail identifying the type of SM or types of stakeholders (e.g. problem owner, nurse practitioner).

Title and abstract screening was done following the criteria in Table A2 below. Full-text screening was done following the criteria in Table A3 below.

**Search Strategy**

The search strategy was developed in consultation with expert librarians in the health sciences and business fields. The search was developed in the OVID platform based around the three main terms *SM*, *health* and *stakeholder engagement*, and tailored for each electronic database as appropriate. Keywords were generated from an initial set of known papers and a scan their reference lists.

Search strategies were developed separately for medical and business electronic databases (see Table A1) as significant disparities were found in the terminology and keywords used. Non-MeSH terms were used as MeSH terms don’t map well for SM in healthcare, a conclusion which has been made by previous similar reviews^2^.

We performed a search of the electronic databases MEDLINE, EMBASE, Scopus, Web of Science and Business Source Complete databases, from their inception through 21 February 2020. The decision about which databases to include was based on the number of known relevant articles captured by the search strategy in each database. Additional candidate studies were identified by reviewing the reference lists of eligible studies.

**Table A1.** Search terms for each database

| **Database** | **Search terms** | |
| --- | --- | --- |
| Web of Science Core Collection | (TI=( simulation* OR "system dynamics" OR "discrete event" OR "agent based") NOT TI=( training OR education OR teaching OR "clinical simulation" OR "medical simulation" ) AND TS=( "group model building" OR "focus group*" OR interview* OR "advisory board" OR "advisory committee" OR "steering group" OR "co-design" OR "co-produc*" OR stakeholder* OR client* OR customer* OR implementation ) AND TS=( "health care" OR "healthcare" OR hospital* OR "primary care" OR "public health" OR "health policy" OR "health service" )) OR (TS=(participatory PRE/2 (model* OR simulation) AND TS=( "health care" OR "healthcare" OR hospital* OR "primary care" OR "public health" OR "health policy" OR "health service")) | |
| OVID: Embase, MEDLINE | 1 | (“health care” or “healthcare” or hospital* or “primary care” or “public health” or “health policy” or “health service”).ab. or (“health care” or “healthcare” or hospital* or “primary care” or “public health” or “health policy” or “health service”).kw. or (“health care” or “healthcare” or hospital* or “primary care” or “public health” or “health policy” or “health service”).ti. |
|  | 2 | (participatory adj2 (simulation or model*)).ab. or (participatory adj2 (simulation or model*)).kw. OR (participatory adj2 (simulation or model*)).ti. |
|  | 3 | 1 and 2 |
|  | 4 | (“group model building” or stakeholder* or client* or customer* or implementation or focus group* or interview* or steering group* or advisory board* or advisory committee* or co-design* or co-produc*).ab. or (“group model building” or stakeholder* or client* or customer* or implementation or focus group* or interview* or steering group* or advisory board* or advisory committee* or co-design* or co-produc*).ti. (“group model building” or stakeholder* or client* or customer* or implementation or focus group* or interview* or steering group* or advisory board* or advisory committee* or co-design* or co-produc*).kw. |
|  | 5 | ((simulation or system dynamics or discrete event or agent based) not (teaching or education or training or clinical simulation or medical simulation)).ti. |
|  | 6 | 1 and 4 and 5 |
|  | 7 | 3 or 6 |
|  | 8 | limit 7 to english language |
| Scopus | ( ( TITLE-ABS-KEY ( "health care"  OR  "healthcare"  OR  hospital*  OR  "primary care"  OR  "public health"  OR  "health policy"  OR  "health service" ) )  AND  ( ( TITLE-ABS-KEY ( "group model building"  OR  "focus group*"  OR  interview*  OR  "advisory board"  OR  "steering group"  OR  "advisory committee"  OR  "co-design*"  OR  "co-produc*" OR stakeholder*  OR  client*  OR  customer*  OR  implementation ) ) )  AND  ( ( TITLE ( simulation*  OR  "system dynamics"  OR  "discrete event"  OR  "agent based" ) )  AND NOT  ( TITLE ( training  OR  education  OR  teaching  OR  "clinical simulation"  OR  "medical simulation" ) ) ) )  OR  ( ( TITLE-ABS-KEY ( participatory  PRE/2  ( model*  OR  simulation* ) ) )  AND  ( TITLE-ABS-KEY ( "health care"  OR  "healthcare"  OR  hospital*  OR  "primary care"  OR  "public health"  OR  "health policy"  OR  "health service" ) ) ) | |
| Business Source Complete | (((((DE "PARTICIPATORY design") OR (DE "ENGAGEMENT letters" OR DE "STAKEHOLDER theory" OR DE "STAKEHOLDER analysis")) OR (DE "FOCUS groups" OR DE "GROUP work in research" OR DE "CUSTOMER advisory boards" OR DE "GROUP decision making" OR DE "SPECIAL interest groups (Associations)" OR DE "GROUP problem solving" OR DE "CROSS-functional teams")) OR (DE "INTERVIEWING" OR DE "QUALITATIVE research")) OR (DE "ADVISORY boards" OR DE "EXECUTIVE advisory bodies")) OR (DE "CHANGE management")  AND  DE "SIMULATION methods & models" OR DE "computer simulation"  AND  (DE "MEDICAL care" OR DE "HEALTH facilities" OR DE "HEALTH services administration" OR DE "HEALTH maintenance organizations" OR DE "HEALTH care reform" OR DE "CONSUMER-driven health care" OR DE "HEALTH & economic development" OR DE "UTILIZATION of health facilities" OR DE "MENTAL health service costs" OR DE "MENTAL health personnel" OR DE "MENTAL health facility employees" OR DE "MEDICARE health maintenance organizations" OR DE "HEALTH services administrators" OR DE "HEALTH insurance reimbursement" OR DE "HEALTH information exchanges" OR DE "HEALTH care management industry" OR DE "HEALTH care industry securities") | |

**Study Selection**

Initial references were collated and duplicates removed, before being imported into Covidence^3^ for screening of titles and abstracts against eligibility criteria. Two independent reviewers (TZ & KL) achieved excellent agreement (Kappa = 0.87) after reviewing 25% of studies (421) in duplicate, so the remaining studies were reviewed by the first author. Disagreements in the initial screening were included for full-text screening. Full-text screening followed an analogous process of an initial 25% duplicate screening, which achieved excellent agreement (Kappa = 0.88). Disagreements in full-text screening were resolved by discussion.

**Table A2.** Title and abstract screening

|  | **Stakeholder Engagement** | **Simulation Modelling** | **Healthcare** |
| --- | --- | --- | --- |
| **Inclusion criteria** | Stakeholders engagement - abstract must refer to:  - Stakeholders are involved in the process before the stage of implementing the model.  - Engagement must occur before computer simulation model is built i.e. during development and building of the model | - computational simulation only  - system dynamics, discrete event simulation and/or agent-based modelling. If type not stated at abstract level, citations will be included. | - Project or case study focussing on a health care/mental health care issue - from epidemiology up to policy level simulation models.  - Simulation models which look at the impact of wider changes on health will be included as long as health/health care is the primary consideration of the model e.g. alcohol, drug misuse, smoking will be included if the impact of changes on health/health care system is primary aim. Disability-related studies are included. |
| **Exclusion criteria** | - Stakeholders are only engaged at the stage of implementation  - Provision of interventions/scenarios to test only  - Validation of model only, unless used to iteratively improve model  - "Expert opinion" used to parameterize the model  - Existing surveys used | - Markov models, montecarlo simulation etc. unless combined with SD, DES or ABM.  - Any non-computational type of simulation e.g. Clinical simulation, high-fidelity simulation, simulation training etc | - Primary aim of model other than health e.g. environmental air pollution, effect of drugs/alcohol on crime.  - Dentistry-related topics are excluded  - Simulation models of medical information storage only are excluded.  - Molecular-level simulations are excluded. |

**Table A3.** Full-text screening

|  | **Stakeholder Engagement** | **Simulation Modelling** | **Healthcare** |
| --- | --- | --- | --- |
| **Inclusion criteria** | Minimum information in full-text to be included is:  1. who the stakeholders were (descriptions such as "problem owner" is sufficient)  2. when they were involved (at what stage of the project the stakeholders were involved)  3. what the function, expected benefit or actual benefit was of the engagement (could be process-driven e.g. "to inform stakeholders about results" or "facilitate social learning" or outcome-driven e.g. "increase acceptance of model") | - computational simulation only  - system dynamics, discrete event simulation and/or agent-based modelling. Must state this at the full-text stage. Hybrid simulation models using a least one of these types will be included - can be combined with another type of modelling. | - Project or case study focussing on a health care/mental health care issue - from epidemiology up to policy level simulation models.  - Simulation models which look at the impact of wider changes on health will be included as long as health/health care is the primary consideration of the model e.g. alcohol, drug misuse, smoking will be included if the impact of changes on health/health care system is primary aim. Disability-related studies are included. |
| **Exclusion criteria** | - Stakeholders are only engaged at the stage of implementation  - Provision of interventions/scenarios to test only  - Validation of model only, unless used to iteratively improve model  - "Expert opinion" used to parameterize the model  - Existing surveys used | - Markov models, montecarlo simulation etc. unless combined with SD, DES or ABM.  - Any non-computational type of simulation e.g. Clinical simulation, high-fidelity simulation, simulation training etc | - Primary aim of model other than health e.g. environmental air pollution, effect of drugs/alcohol on crime.  - Dentistry-related topics are excluded  - Simulation models of medical information storage only are excluded.  - Molecular-level simulations are excluded. |

**Data Extraction**

Included studies had data extracted using a standardized form (Table A4), which was piloted using a small sample (n=10) of randomly selected studies by two independent reviewers to assess reliability, with any discrepancies discussed and resolved prior to full data extraction by TZ. Data extracted consisted of general details (e.g. year published, publication type, country of study, topic area), data relating to the simulation model (e.g. software used, type of simulation, main output measure), and data relating to the participatory process (e.g. number and types of stakeholders engaged, recruitment and sampling of stakeholders, modes of engagement, activities used to involve stakeholders and impacts of engagement). We also captured author’s recommendations or reported enablers for facilitating stakeholder engagement.

Topic areas were categorised as *Health and Care Systems Operation*, *Epidemiology, Health Promotion and Disease Prevention*, *Health and Care Systems Design, Medical Decision Making* or *Extreme Events Planning*^4^. Data relating to the participatory process and impacts were extracted in their original form from study manuscripts, and abstraction only performed at the analysis stage to ensure the contextual aspects of the data were captured.

**Table A4.** Data extraction template

**General details:**

| Author(s) |  |
| --- | --- |
| Year |  |
| Title |  |
| Source |  |
| DOI |  |
| Publication Type |  |
| Country |  |

**Setting and Simulation details:**

| Setting | E.g. hospital, community, company |
| --- | --- |
| Topic Area | - Epidemiology, disease prevention & screening - Medical decision making and treatment evaluation - Healthcare system operations (Resource optimisation) - Healthcare system design and planning |
| Research Question | - Quoted - Description |
| Type of simulation | - DES - ABM - SD - Hybrid: describe |
| Software/Language used | E.g. AnyLogic, SIMUL8, C++ model… |
| Interventions/Scenarios Tested | E.g. Adding 1 nurse, changing policy on alcohol taxation… |
| Key Simulation Output | E.g. waiting time, LOS… |
| Recommendations from simulation | E.g. should add another nurse before investing in more beds |

**Stakeholder Engagement details:**

| Approach/methodology for stakeholder engagement incl. terminology | E.g. "participatory modelling", "fully-facilitated DES"… |
| --- | --- |
| + details about methodology if given | - Existing, adapted, or novel? - Derived from which field? |
| Types of stakeholders involved incl. terminology | E.g. patients, nurses, senior consultants, "problem owners"… |
| When were stakeholder engaged? | E.g. during problem structuring, during implementation stage… |
| What activities did the stakeholders get involved with? | E.g. Providing investigational components/scenarios to test, contributing mental model of system to the conceptual model… |
| Modes of engagement | E.g. interview, survey, focus group … |
| Mechanisms of interaction | E.g. webinar, face-to-face … |
| Special provision for patients if involved? | E.g. extra information given about the topic, incentives provided… |
| Freq/duration of contact | E.g. 3 workshops one 4hrs each |
| Dissemination method | E.g. Delphi method, shared decision-making, qualitative synthesis, quantitative survey … |
| Influence of stakeholders on model purpose, design or structure | E.g. Since end-users have no influence on X part of the system, this was only modeled in a simplified way |
| Expected/intended positive outcomes of stakeholder engagement | - Quoted - Framework from Seidl, 2015 |
| Reported positive outcomes of stakeholder engagement | - Quoted - Framework from Seidl, 2015 |
| Reported negative outcomes of stakeholder engagement |  |
| Reported enablers of engagement of stakeholders |  |
| Reported barriers to engagement of stakeholders |  |
| Implementation Level | - Theoretical (proposed by authors) - Conceptualized (discussed with client organisation) - Implemented (changes attempted within organisation on the back of simulation study) |
| Success reported based on four stage model of success (Robinson & Pidd, 1998) | - Stage 1: The study achieves its objectives and/or shows a benefit; - Stage 2: The results of the study are accepted; - Stage 3: The results of the study are implemented; - Stage 4: Implementation proved the results of the study to be correct |
| Successful elements of implementation and enablers |  |
| Unsuccessful elements of implementation and barriers |  |
| Other lessons learned |  |
| Resources associated with stakeholder engagement activities |  |

**Data Analysis**

Data extracted from included studies were analyzed using summary statistics, a combination of deductive and inductive content analysis^5^ and narrative synthesis elements^6^. Summary statistics were used to analyze study characteristics. Content analysis was applied to synthesis qualitative data describing the participatory process and intended or reported impacts of this process. We used matrices to explore the overlap between process characteristics and intended or reported impacts, in order to map how the nature of the process may link to impacts. To obtain a richer understanding of the participatory processes, a narrative synthesis approach was used to analyze the role of stakeholder engagement activities within the SM process.

The types of stakeholders were categorized according to an adaptation of the 7P’s framework, with purchasers and payers combined into a single category. The generic stages of SM lifecycles were used to represent modeling stages that stakeholders could be engaged in – problem formulation, conceptual modeling, computer modeling, model verification and validation, experimentation and implementation^7^. Other stages which engaged stakeholders that didn’t fit into the generic stages were inductively coded. The intended and reported benefits of the participatory process were coded within a framework adapted from Barreteau et al. comprising three broad types of benefits for (1) the design of the model, (2) the implementation of the model, and (3) the stakeholder participants^8^. Within this framework, inductive content analysis was used to identify and quantify the sub-groups of benefits.

This synthesis concentrated on authors’ reporting of the participatory process in SM studies, which meant that outcome measures from the studies were not included. Therefore, no formal assessment of risk of bias was necessary either in individual studies or across studies^9^.

**Appendix B: Additional details on Stakeholder Participants**

***Stakeholder Types***

The type of stakeholders involved in the participatory process varied widely between studies. The most frequently engaged stakeholders were Providers (n=23). Of the studies engaging Providers, most engaged clinicians (n=21) or clinical management staff (n=19) in the institution or system under enquiry. Clinicians engaged were frequently stated to have specific experience in the problem and often had various additional roles, such as working with public health agencies^10^ or as part of hospital strategy teams^11^, so their individual involvement brought multiple perspectives to the team. In one study^12^, the simulation modeler was a clinician and health informatician in the area of practice specific to the problem. Other stakeholders within the Provider category brought relevant technical skills and experience, including analysts with quantitative skills including simulation^13^ and case managers or financial directors with strategic and operational experience^14^. Policy-makers (n=7), Purchasers/Payers (n=9) and Patients and the Public (n=6), were engaged in a similar number of studies. The median number of stakeholder types engaged was 2, though the mode was only 1 type of stakeholder. There were a minority of studies which engaged a more diverse set of stakeholder types, four studies engaged 3 different types of stakeholders and one study engaged 4. Each study on average engaged 2 different types of stakeholder participants.

***Stakeholder Numbers***

The number of individual stakeholders engaged during the process was reported in 50% (n=14) of studies. Fewer studies (n=5) reported the number of stakeholders who participated in specific engagement activities. Of those that did report the number of stakeholders involved, there was large variation between studies. Some studies involved less than 5 stakeholders throughout the process of simulation model building, whilst others involved over 300, with the median being 26 participants.

***Stakeholder Recruitment***

Recruitment of stakeholders was typically done by accessing employees or existing affiliates through the institution under study, and in some studies the SM was part of a larger study or initiative. In one study^15^, the local leadership of the institution under study offered “workload credit” to relevant employees to volunteer in the participatory modeling process. Stakeholder incentives for participation were not discussed in any other studies. As a sampling technique, purposive sampling was most common (n=8), in particular for studies involving a smaller number of participants. Other stakeholder identification or sampling techniques used were actor inheritance^16^, snowball sampling^17^, random sampling^18^ and convenience sampling^18,19^. Only 3 studies specified inclusion criteria for stakeholder participants^18-20^.

**Appendix C: Additional details on** **the Participatory Process**

***Stages of Engagement***

Inductive coding of stages that didn’t fit the generic framework mapped to three additional stages – parameterization or data collection, recruitment of other participants, and process evaluation. The inclusion criteria required engaged stakeholders’ input to influence model design, therefore all studies engaged stakeholders during the conceptual model building stage in some form. Despite this, all but one study^21^ engaged stakeholders in another stage of the SM process. Common stages of engagement aside from conceptual model building were problem formulation (74%), model verification & validation (74%) and experimentation & implementation (59%). It was relatively uncommon for studies to engage stakeholders during the computer model building stage (11%), however, collecting data or parameters through the engagement of stakeholders was used by almost half the studies as a means of quantifying the computer model (41%). Only two studies (7%) engaged stakeholders in evaluating the participatory process in the simulation study. In 9 studies (33%), stakeholders were engaged in all the generic stages (from the beginning to the end of the SM lifecycle – excluding computer model building).

***Modes of Engagement & Facilitation***

There were four primary modes employed by studies in the engagement of stakeholders: discussion/dialogue (n=13, 48%), interviews (n=11, 41%), workshops (n=8, 30%), and meetings (n=7, 26%). Other engagement modes used were webinars (n=2, 7%), questionnaires (n=2, 7%), focus groups (n=1, 4%), and feedback from public display of the model (n=1, 4%). Modes of engagement varied across the SM stages. The use of discussion/dialogue and meetings was fairly consistent across stages. Interviews were mostly used earlier in the SM process for problem formulation (n=7, 26%) and conceptual modeling (n=5, 19%) while workshops were mostly used in the mid and late stages of the process, for conceptual model building (n=6, 22%), validation & verification (n=6, 22%) and experimentation & implementation (n=5, 19%).

Some studies provided descriptions about how they facilitated this input, which ranged from structured and active methods where stakeholders were asked specific questions^19^ or engaged in purposeful storytelling exercises,^22^ to unstructured and passive methods where stakeholders provided feedback about or annotated an existing model^13,23^. More structured methods of facilitation were used in early stages when studies were engaging stakeholders in designing the model from scratch^15,19,22,24-27^, and more passive methods were used when stakeholders were engaged at a later stage and a draft model had already been designed^11,13,21,23,28,29^.

The type of input sought from the stakeholders, and the role of their engagement in model design was similar amongst studies which described this, which was to provide (1) the *variables* or *factors* which they considered important in the problem, and (2) the *relationships* or *causal* *pathways* they know or perceive between these variables. However, there was some variability in the terminology used by different studies to describe this, including: *‘provide expert opinion in the events involved in…’*^21^*, ‘provide evidence for model elements…’*^16^*,* and *‘identify features of… that might be important in…’*^26^*, ‘identify key causal relationships that impacted …’*^27^*, ‘jointly conceptualise and collaboratively maps the ‘systems’*^22^*, ‘collaboratively map the key risk factors and likely causal pathways…’*^24^, and *‘in depth deliberation to map a shared mental model of the causal pathways for the focus issue, and the mechanisms by which …’*^30^*.*

Another aspect of facilitation activity which differed between studies was how the products of engagement (inputs from stakeholders) were translated into changes in model design. There were two broad approaches to this. One approach used by the majority of studies was direct model interaction which involved hands-on interaction with the model, where the model itself acts as a communication vehicle (n=18, 67%). This allowed stakeholders to physical manipulate and “play” with the model design to align it with their own mental models about the system in question. Another approach used by studies not involving direct model interaction was to use specific qualitative methods to collect data from stakeholders and incorporate findings into the model design. The most common qualitative analysis method used was thematic analysis (n=4, 15%)^10,18,22,25^. Other formal methods used were qualitative comparative analysis and content analysis on interview transcripts^19^, value coding using a 5-point Likert scale relating to perceived importance and feasibility of specific statements^25^, qualitative formative evaluation of stakeholder concerns and modeling goals^15^, and mediated modeling “codification” of stakeholder knowledge from interviews^19^.

# Appendix D: Additional details on the Impacts of Stakeholder Engagement

## Impacts of participatory process on model design

There were four types of impacts reported from stakeholder engagement on model design: (1) increased relevance of the problem addressed (n=4, 14%), (2) better quality/accuracy of the model for its purpose (n=9, 33%), (3) improved identification or access to better data (n=4, 15%), and (4) expertise from a range of perspectives (n=9, 31%).

Breaking these impacts down into the different stages of SM, the participatory process was reported to impact model design during various stages. Participation was reported as a means to increase the relevance of issues addressed during problem formulation, and to focus only on the most important real-world questions to be explored^15,18,22,31^. Baldwin et al. state this explicitly, reporting that the use of a participatory process allows the modeler to better develop a model that fits “*the purpose as defined by the stakeholders and not that as perceived by the modeler*.”^32^

During the conceptual modeling phase, several authors noted that the participation of stakeholders allowed robust and transparent assumptions to be made such that the accuracy of the model was better aligned to its purpose^11-14,16,22-24,28^. The inclusion of diverse stakeholders in the participatory process was reported to both necessitate^26^ as well as facilitate the use of simplifying assumptions in models. Nine studies mentioned that involving stakeholders account for a range of perspectives^17,19,24,26,27,29,32-34^. Freebairn et al. discusses this benefit of engagement over traditional analytical research methods, stating that a “*multi-disciplinary group of health sector participants brought to modeling discussions a breadth and depth of knowledge and rich experience regarding the issue that would be impossible to gain from reviewing the data/literature alone*”^22^.

As well as combining a diverse knowledge base during model-building, involving stakeholders improved data collection and parameterization as they provided access to rare empirical data or identified relevant evidence sources to help face the challenge of quantifying the model^12,15,22,24^. Expert stakeholders were also able to identify limitations in the data and published literature^22^.

## Impacts of participatory process on model implementation

There were three types of impacts reported from stakeholder engagement on model implementation: (1) refined use of terminology (n=1, 3%) ^25^, (2) greater acceptance or ownership of the model (n=5, 17%), and (3) improved implementation or suitable use of the model (n=8, 27%).

Involving stakeholders in model design process, by increasing the acceptance and ownership of the model^13,22,24,28,34^, may have impacts further downstream on the implementation of simulation findings^13,15,24,29,33-36^. For instance, Bowers et al. reported that collaborative model design process *“created a genuine sense of ownership of the changes”* when simulation findings were implemented, and that this participatory process is “*an excellent environment for exploring the use of simulation as an embedded support tool in healthcare redesign”*^13^. Similarly, stakeholders engaged in Freebairn et al. stated that the *“co-production aspect”* engendered *“trust in the model and its outputs as a decision-support tool”*^33^*.* Lane et al., despite initial difficulties with buy-in from the gatekeeping stakeholder, ultimately stated that *“she definitely [had] a feeling of ‘model ownership’!”*^28^*.* Baldwin et al. reasons that there a link between the *“greater involvement and authority”* of decision-makers in the SM process, and the *“a higher possibility that findings from the modeling will be implemented”*, due to increased ownership of the problem and its eventual solution^32^.

In the overlap between the participatory process and impacts, the benefits of acceptance or ownership of the model and productive discussion or shared understanding were strongly associated with the involvement of direct model interaction as part of studies’ participatory processes. Studies involving direct model interaction were more likely to report benefits for stakeholder ownership of the model (56% vs 11%) and productive discussion & shared understanding (61% vs 22%), compared to studies which did not involve direct model interaction.

The steps of generating and experimenting with alternative scenarios or interventions in the model were reported to be improved by the engagement of stakeholders, as the “*expertise of the participant group grounded the model in the real-world experience of intervention effectiveness*”^22^. Therefore, the participatory modeling not only increases the understanding of the current system, it enables the collaborative designing of improved systems through experimentation with, and implementation of, the model.

## Impacts of participatory process on stakeholder participants

There were three types of impacts reported from the participatory process on the stakeholder participants: (1) productive discussion or shared understanding of the problem (n=8, 28%), (2) “learning” (n=5, 17%), and (3) better problem solving or decision-making (n=10, 35%).

Multiple studies referred to the function of the simulation model in enabling more productive discussion and *“allowing needed dialogue”*^15^ between stakeholders, particularly those from different disciplines^18,22-25,32^. The fact that models require perspectives and assumptions to be made explicit in a graphical representation which imitates the real system meant that several studies found that stakeholders could communicate effectively using the model^18,24,30,32^. Freebairn et al. state that the collaborative process of model design *“enhanced each participant group’s understanding of the others’ knowledge”*^30^*.* Baldwin et al. builds on this, proposing that *“modeling is actually a conversation between the model and the stakeholders, thus helping to enhance the communication between the different stakeholders involved”* ^32^*.* From the perspectives of the stakeholder participants, *“[they] often commented on the value of seeing the whole system in its entirety”*^23^.

In addition to enabling productive discussion and a shared understanding between stakeholder participants, the participatory process was reported to in five studies to enable “learning” by stakeholders^15,24,28,30,33^. The type of “learning” enabled was not discussed by Lane et al.^28^ or Zimmerman et al.^15^, while Atkinson et al. specifically identifies *“learning about complex problems”*^24^. In their studies, Freebairn et al. identify different mechanisms via which “learning” occurred, where *“participants were learning from each other”*^33^, and *“two-way learning”* occurred between the model architecture and stakeholders’ understanding of the problem^30^.

The impact of the participatory process as better problem solving or decision-making^11,15,17,18,28,29,33,36^ ultimately links to the improved implementation of the model, which due to the engagement of relevant stakeholders is *“better aligned to the decision-support needs of policy makers”*^22^. During the process evaluation conducted by Freebairn et al., the outcome of the participatory process to “produce an innovative decision-support tool” was *“highly valued by most [stakeholder] interviewees”*^33^*.*

One study reported the impact of the participatory process for improving the process itself – “the longer-term relationship with the client provided a good opportunity to gain honest feedback and to redress some of the problems encountered in the implementation of this study in subsequent simulations”^13^.

**Appendix E: Additional details on the Specific Processes Used to Engage Stakeholders**

Some studies provided specific details about the process used to combine stakeholder engagement and SM and how to do this well which can provide practitioners and decision-makers as well as researchers with useful guides for engaging in such processes^16,18,20,22,32^.

Bell et al. discusses the use of an “industry-as-laboratory” research paradigm in order to (a) solve specific problems, (b) support both process and artifact analysis and (c) allow iterative, interpretive approaches to upfront hypothesis^16^. Specific focus is given to the process of using collaborative SM in building a hybrid model (two different types of simulation models which are integrated). They outline the “Collaborative Hybridization Process” which is broken down into Scoping, Evidence Gathering, Individual Modeling, Collaborative Modeling, Scenario Execution (i), Demonstration, Interim Review, Acceptance Testing (including model walkthrough), Scenario Execution (ii), Reporting, Final Review. The authors discuss design considerations relevant for stakeholder engagement at two key transition points between process steps. Firstly, at the Scoping-Evidence Gathering transition, the authors use a 3-step scoping process – (1) contract negotiation, 2) kick-off meeting and 3) scoping documentation – to ensure a clear understanding of the project aims, objectives and key stakeholder groups before stakeholder interviews for evidence gathering begins. Actor Inheritance is used to identify relevant stakeholders and their organizational units. Secondly, at the Individual-Collaborative Modeling transition, the authors identify design considerations for appropriately visualising a hybrid simulation model when working “across organizational boundaries”. More specifically, they discuss how the process of producing “state machine diagrams” of hybrid models can be used to support collaborative working and decision making.

Rwashana et al. adapted the dynamic synthesis methodology to the healthcare context in an iterative 6-stage framework comprising problem statement, field studies, model building, case study simulations and policy analysis^18^. The framework outlines the methods and objectives of each stage of the process and the authors provide narrative descriptions about how this was carried out in the case context of this study. Therefore, this study provides narrative detail about the process applied to a specific use-case but detailed generalizable guidelines are not provided beyond for the 6-stage framework.

Freebairn et al. provides two frameworks to guide the process of engaging stakeholders in SM^22^. The first framework outlines the activities involved in the participatory process, broken down into 3 separate workshops which engage stakeholder participants as well as ongoing core model building group activities. The authors also provide narrative descriptions about how the process was carried out, highlighting that contact with stakeholders was initiated early and the scope of engagement negotiated to include key policy and planning questions, the interaction of key risk factors, and context specific intervention priorities. The process was also carried out such that participants had differing levels of involvement (intensity and duration) in the project. Some were workshop participants only whilst others contributed as workshop facilitators, attended project team meetings, and were involved in subsequent communications about the application of the simulation model. The second framework developed by the authors provides an overview of the analytical objectives and decision-making processes involved in the participatory development of a simulation model, including the challenges and opportunities of engaging stakeholders. Freebairn et al.^20^ describes this same protocol used for the Freebairn et al.^22^ study, and details examples of semi-structured interview questions used to obtain stakeholder views both pre-workshop for scoping the problem as well as post-workshops for evaluation of the participatory process from stakeholders’ perspectives.

Baldwin et al. discusses a “soft approach” to simulation in healthcare management which engages stakeholders in the process, called Modeling Approach that is Participatory Iterative for Understanding (MAPIU)^32^. The authors provide a classification system for stakeholders as *problem owners*, *experts* and *actual users*, and describe what each stakeholder type contributes to the process. They also provide a framework to show how different stakeholder contributions fit into the overall structure of the MAPIU process. The process in MAPIU is broken by the authors into *modeling* and *communication* which is particularly important to link the stakeholder participants in the process. *Communication* is further broken down into *stakeholder-to-model* communication (in which the model is either a destination where requirements and needs are fed into or a source where information is retrieved from) and *stakeholder-to-stakeholder communication* (in which the model could be used as a means of communication but not a source or a destination). An example is provided of SM in an evaluative study of the cost-effectiveness of a liver transplantation technology, however, the authors state that the MAPIU approach is “adaptive to changing requirements” meaning that it provides guidelines and principles which are flexible to the particular use-case of the SM.

# Appendix References

1. Badland H, White M, MacAulay G, et al. Using simple agent-based modeling to inform and enhance neighborhood walkability. *International Journal of Health Geographics.* 2013;12(1):58.

2. Chen J. *Agent-based Modelling in Healthcare Operations: A Systematic Review of its Scope, Quality and Implementation*, University of Oxford; 2018.

3. *Covidence systematic review software* [computer program]. Melbourne, Australia: Veritas Health Innovation.

4. Mielczarek B, Uziałko-Mydlikowska J. Application of computer simulation modeling in the health care sector: a survey. *SIMULATION.* 2010;88(2):197-216.

5. Elo S, Kyngäs H. The qualitative content analysis process. *Journal of Advanced Nursing.* 2008;62(1):107-115.

6. Popay J, Roberts H, Sowden A, et al. *Guidance on the conduct of narrative synthesis in systematic reviews: A product from the ESRC methods programme.* 2006.

7. Yapa ST. *A structured approach to rapid simulation model development*, Sheffield Hallam University; 2006.

8. Barreteau O, Bots P, Daniell K, et al. Participatory Approaches. In: Edmonds B, Meyer R, eds. *Simulating Social Complexity: A Handbook.* Berlin, Heidelberg: Springer Berlin Heidelberg; 2013:197-234.

9. Domecq JP, Prutsky G, Elraiyah T, et al. Patient engagement in research: a systematic review. *BMC Health Services Research.* 2014;14(1):89.

10. Barbrook-Johnson P, Badham J, Gilbert N. Uses of Agent-Based Modeling for Health Communication: the TELL ME Case Study. *Health Communication.* 2017;32(8):939-944.

11. Glasgow SM, Perkins ZB, Tai NRM, Brohi K, Vasilakis C. Development of a discrete event simulation model for evaluating strategies of red blood cell provision following mass casualty events. *European Journal of Operational Research.* 2018;270(1):362-374.

12. Johnson O, Hall P, Hulme C, Johnson OA, Hall PS. NETIMIS: Dynamic Simulation of Health Economics Outcomes Using Big Data. *PharmacoEconomics.* 2016;34(2):107-114.

13. Bowers J, Ghattas M, Mould G. Success and failure in the simulation of an Accident and Emergency department. *Journal of Simulation.* 2009;3(3):171-178.

14. Giesen E, Ketter W, Zuidwijk R. An agent-based approach to improving resource allocation in the dutch youth health care sector. *17th European Conference on Information Systems, ECIS 2009.* 2009.

15. Zimmerman L, Lounsbury DW, Rosen CS, Kimerling R, Trafton JA, Lindley SE. Participatory System Dynamics Modeling: Increasing Stakeholder Engagement and Precision to Improve Implementation Planning in Systems. *Administration and Policy in Mental Health and Mental Health Services Research.* 2016;43(6):834-849.

16. Bell D, Cordeaux C, Stephenson T, et al. Designing effective hybridization for whole system modeling and simulation in healthcare. *2016 Winter Simulation Conference, WSC 2016.* 2016;0:1511-1522.

17. Uebelherr JM, Hondula DM, Johnston EW, et al. Innovative participatory agent based modeling using a complexity governance perspective. *16th Annual International Conference on Digital Government Research, dgo 2015.* 2015;27-30-May-2015:307-308.

18. Rwashana AS, Nakubulwa S, Nakakeeto-Kijjambu M, Adam T. Advancing the application of systems thinking in health: understanding the dynamics of neonatal mortality in Uganda. *Health Research Policy and Systems.* 2014;12:36.

19. de Andrade L, Lynch C, Carvalho E, et al. System dynamics modeling in the evaluation of delays of care in ST-segment elevation myocardial infarction patients within a tiered health system. *PLoS ONE [Electronic Resource].* 2014;9(7):e103577.

20. Freebairn L, Atkinson JA, Kelly P, McDonnell G, Rychetnik L. Simulation modelling as a tool for knowledge mobilisation in health policy settings: a case study protocol. *Health Research Policy and Systems.* 2016;14.

21. Hung GR, Whitehouse SR, Oneill C, Gray AP, Kissoon N. Computer modeling of patient flow in a pediatric emergency department using discrete event simulation. *Pediatric Emergency Care.* 2007;23(1):5-10.

22. Freebairn L, Atkinson JA, Osgood ND, Kelly PM, McDonnell G, Rychetnik L. Turning conceptual systems maps into dynamic simulation models: An Australian case study for diabetes in pregnancy. *PLoS ONE.* 2019;14(6).

23. Lattimer V, Brailsford S, Turnbull J, et al. Reviewing emergency care systems I: insights from system dynamics modelling. *Emergency Medicine Journal.* 2004;21(6):685-691.

24. Atkinson JA, O'Donnell E, Wiggers J, et al. Dynamic simulation modelling of policy responses to reduce alcohol-related harms: rationale and procedure for a participatory approach. *Public Health Research & Practice.* 2017;27(1).

25. Hassmiller Lich K, Urban JB, Frerichs L, Dave G. Extending systems thinking in planning and evaluation using group concept mapping and system dynamics to tackle complex problems. *Evaluation and Program Planning.* 2017;60:254-264.

26. Homa L, Rose J, Hovmand PS, et al. A Participatory Model of the Paradox of Primary Care. *Annals of Family Medicine.* 2015;13(5):456-465.

27. Matchar DB, Ansah JP, Bayer S, et al. Simulation modeling for primary care planning in Singapore. *2016 Winter Simulation Conference, WSC 2016.* 2016;0:2123-2134.

28. Lane D, Monefeldt C, Husemann E, Lane DC. Client involvement in simulation model building: hints and insights from a case study in a London hospital. *Health Care Management Science.* 2003;6(2):105-116.

29. Rosmulder R, Krabbendam J, Kerkhoff A, Houser C, Luitse J. Computer Simulation Within Action Research: A Promising Combination for Improving Healthcare Delivery? *Systemic Practice & Action Research.* 2011;24(5):397-412.

30. Freebairn L, Rychetnik L, Atkinson JA, et al. Knowledge mobilisation for policy development: implementing systems approaches through participatory dynamic simulation modelling. *Health Research Policy and Systems.* 2017;15.

31. Baldwin LP, Eldabi T, Paul RJ, Burroughs AK. Using simulation for the economic evaluation of liver transplantation. *Proceedings of the 32nd conference on Winter simulation.* 2000:1963–1970.

32. Baldwin LP, Eldabi T, Paul RJ. Simulation in healthcare management: a soft approach (MAPIU). *Simulation Modelling Practice and Theory.* 2004;12(7-8):541-557.

33. Freebairn L, Atkinson JA, Kelly PM, McDonnell G, Rychetnik L. Decision makers' experience of participatory dynamic simulation modelling: methods for public health policy. *BMC Medical Informatics and Decision Making.* 2018;18.

34. Roberts N, Li V, Atkinson JA, et al. Can the Target Set for Reducing Childhood Overweight and Obesity Be Met? A System Dynamics Modelling Study in New South Wales, Australia. *Systems Research and Behavioral Science.* 2019;36(1):36-52.

35. Mackay M, Qin S, Clissold A, et al. *Patient flow simulation modelling - an approach conducive to multi-disciplinary collaboration towards hospital capacity management.* 2013.

36. Uriarte AG, Zúñiga ER, Moris MU, Ng AHC. System design and improvement of an emergency department using Simulation-Based Multi-Objective Optimization. *31st Euro Mini Conference on Improving Healthcare: New Challenges, New Approaches.* 2015;616.
