# Supplementary material for: Engaging Healthcare Staff and Stakeholders in Healthcare Simulation Modeling to Better Translate Research Into Health Impact: A Systematic Review
Source: Front Health Serv. 2021 Nov 23;1:644831. doi: 10.3389/frhs.2021.644831 (PMC10012644; doi:10.3389/frhs.2021.644831)
Supplement: Supplementary file 3 [file Data_Sheet_3.docx]

| 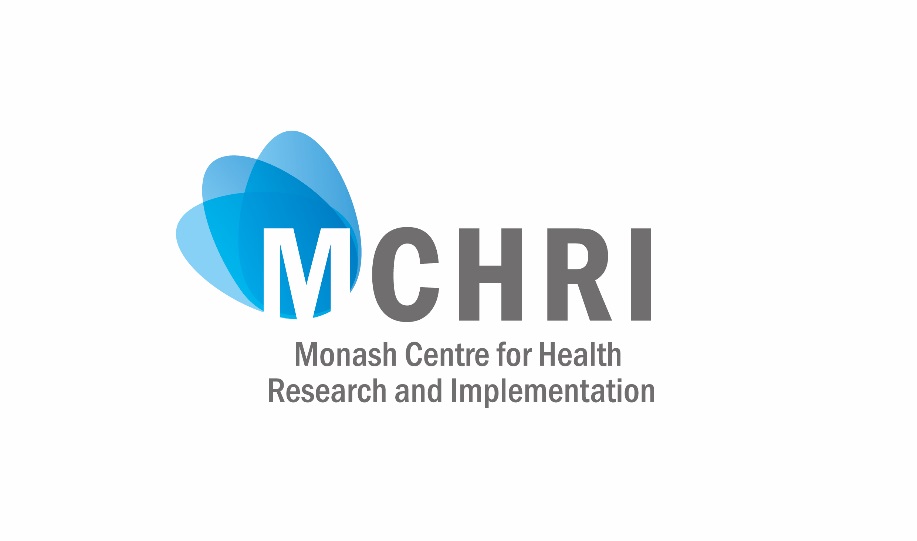 | **Protocol** |
| --- | --- |
|  | **A Systematic Review of Stakeholder Involvement in Health Care Simulation Modelling** |
|  | Thea Zabell, Katrina Long, Joanne Enticott, Ian McLoughlin, Bengianni Pizzirani |
|  | February 2020 |

**Background**

Health systems are complex adaptive systems that are characterised by a diverse range of stakeholders, relationships and processes. Phenomena of these systems such as interdependencies, feedback loops, non-linearity, and emergence underlie the difficulties in using traditional analytical approaches to tackle complex health system problems. As a means of representing this complexity, simulation modelling can be used to explore these systems, wherein different agents and resources interact to elicit emergent system behaviours which may be unexpected at the level of individual components. Simulation modelling involves ‘experimentation with a simplified imitation (on a computer) of an operations system as it progresses through time, for the purpose of better understanding and/or improving that system’. As a safe virtual testbed, it is a medium for assessing the potential impacts of decisions in a simulated environment. The counterfactual reasoning capabilities of simulation modelling allows ‘what if’ scenarios to be interrogated in the simulated environment for a range of purposes including resource allocation, hospital and location planning, organizational redesign, policy-making and more. Thus, simulation modelling has the potential to improve the evidence-base for decision-making and to support the implementation of effective practices in health systems.

It is a vital and pragmatic tool that has been leveraged relatively recently in the health care industry but remains scarcely adopted so the full potential of simulation modelling for improving health systems is yet to be realised. Despite a rapidly expanding base of academic literature on simulation modelling in health care, evidence of implementation remains scarce in comparison to other industries using the tool. Whilst the end-goal of simulation modelling research should logically be to mitigate the problems which motivated its commissioning, most such research fails to ever translate its findings into practice. Many simulation studies do not address “customer success” as defined by Robinson & Pidd (1998), and thus ignore the acceptance, implementation and evaluation components of the simulation models and their recommendations. By delivering to their own specifications and failing to align their goals, simulation modellers may overlook what really matters to different stakeholders. In particular, simulation modelling often neglects the central purpose of health care research which is to conceive or deliver meaningful benefits for consumers/patients.

Integrating different perspectives, perceptions and values of stakeholders in health care is one of the major challenges for research relating to management and policy-making, outlined by an ancient Hindu parable of the 6 blind men and the elephant. Herein, "each man describes only the part of the elephant he is touching, forming an incomplete representation of the whole". This illustrates human tendency to “claim absolute truth based on their limited, subjective experience as they ignore other people's limited, subjective experiences which may be equally true”. The recognition of this challenge and the need for decision-making relevant to clinicians, patients and other end-users has shaped the contemporary discourse of stakeholder engagement and consumer and community involvement in health care research. Simulation modelling is a valuable platform for communication that is synergistic with a stakeholder engagement as the process itself fosters dialogue between parties involved. Indeed, it is thought that participatory simulation methods and interventions can be considered a form of ‘learning health system’, where continuous improvement and social learning in healthcare organizations is achieved. There is a wealth of such expected benefits from increased engagement, however many researchers have made the case for developing the currently insubstantial evidence base behind engagement of key stakeholders and end-users in research (Workman et al. 2013, Esmail et al. 2015). This systematic review seeks to address this gap in knowledge and to contribute to the evidence-base behind stakeholder engagement in healthcare simulation modelling by finding out: (1) What approaches to engaging stakeholders in simulation modelling research exist in healthcare settings? (2) What do we know about the effectiveness of these existing approaches in terms of knowledge translation? How does this differ from expected benefits of stakeholder engagement in simulation modelling? (3) What terminology is used to describe stakeholder engagement in simulation modelling research in healthcare settings? How does this compare between business & management journals and clinical journals?

**Objectives**

The aim/s of this systematic review is/are to:

- What methods are used to involve stakeholders in simulation modelling for health?
- What are the functions, benefits, and influences of this on stakeholder involved and on the model and its use?

**Methods**

**Systematic review team**

| **Role** | **Name** | **Declaration of potential competing interests** |
| --- | --- | --- |
| Systematic reviewer | Thea Zabell |  |
| Systematic reviewer | Katrina Long |  |
| Researcher/ Advisors | Bengianni Pizzirani, Ian McLoughlin, Joanne Enticott |  |

This systematic review has been funded by *Eastern Health & Monash University*.

**Eligibility criteria**

Eligibility was contingent upon three elements being present in the study: (1) a primary focus on improving health (2) dynamic simulation modelling to achieve this goal, and (3) engagement of key stakeholders to help build the simulation model. The specific inclusion and exclusion criteria for each of the elements is outlined in Table 1. Only studies with a full-text in English were considered for inclusion. The year of publication was not limited. In general, studies in which stakeholders were actively engaged in designing and conceptualizing the model at the non-computational stage were sought. Studies utilizing only surveys and/or individual interviews were included if their main reported purpose was obtaining stakeholders values, preferences and mental models that related to conceptualization or design of the model.

**Search methods**

**Databases to be searched**

The following electronic databases will be used to identify relevant published literature:

- **Ovid MEDLINE(R) and Epub Ahead of Print, In-Process & Other Non-Indexed Citations, Daily and Versions(R) 1946 to February 21, 2020**
- **Embase Classic+Embase 1947 to 2020 February 21**
- **Scopus**
- **Web of Science Core Collection**
- **Business Source Complete**
- **Search of the bibliographies of relevant reviews identified by the search strategy for identification of additional studies.**

**Search strategy**

Based on the selection criteria and a set of relevant articles, a selection of keywords was decided on. A systematic search was developed using the OVID platform and translated to other databases as appropriate. The decision about which databases to include was based on the number of gold set articles captured by the search strategy in each database. Scopus achieved the best coverage of gold set papers. MEDLINE and Embase achieved less coverage; the MEDLINE database only indexed 50% of the gold set papers. This demonstrated the need for unconventional systematic review searching strategies for healthcare simulation modelling research, in particular for research relating to stakeholder engagement. PsychINFO retrieved no literature according to the search strategy in OVID, illustrating the lack of this type of work within the field of psychology and mental health. Non-MeSH terms were used to search the OVID platform as MeSH terms don’t map well for simulation modelling in healthcare, a conclusion which has been made by previous similar reviews (Chen, 2018). Keywords were generated by reading relevant literature and based on a gold set of papers and a scan of their reference lists for other relevant literature. This was done separately for medical journals and business source complete as significant disparities in the terminology was found. Business source complete Subject Terms were found to match the search criteria and capture relevant literature with much higher specificity than MeSH. Therefore, Subject Terms were used for searching Business Source Complete. The search strategy will be limited to English language papers only.

**Screening of search results**

Covidence will be used to manage search results. Two independent reviewers will review the titles and abstracts of 25% of articles retrieved by the search strategy according to the selection criteria. Full text of the articles will be retrieved for further assessment if the information given suggests that the study meets the selection criteria or if there is any doubt regarding eligibility of the article based on the information given in the title and abstract. In some cases, there may be more than one article describing the same study and reporting different outcomes, in which case this will be recorded, and the data combined at the stage of analysis. Whilst exploring the literature on this topic, one such study was observed to have been reported in two separate journals; a report on the engagement of stakeholders (“client involvement”) in *Health Care Management Science* and a technical report about the simulation model in *Journal of the Operational Research Society*.

**Data extraction**

Data for outcomes according to the selection criteria will be extracted from included studies using a specially developed data extraction form. Information will be collected on general details (title, authors, reference/source, country, year of publication, setting). Where a study is reported as a methodological protocol only from January 2017 onwards, the authors will be contacted regarding whether any empirical studies were conducted using the protocol. Any disagreement will be resolved by discussion to reach a consensus.

**General details:**

| Author(s) |  |
| --- | --- |
| Year |  |
| Title |  |
| Source |  |
| DOI |  |
| Publication Type |  |
| Country |  |

**Setting and Simulation details:**

| Setting | E.g. hospital, community, company |
| --- | --- |
| Topic Area | - Epidemiology, disease prevention & screening - Medical decision making and treatment evaluation - Healthcare system operations (Resource optimisation) - Healthcare system design and planning |
| Research Question | - Quoted - Description |
| Type of simulation | - DES - ABM - SD - Hybrid: describe |
| Software/Language used | E.g. AnyLogic, SIMUL8, C++ model… |
| Interventions/Scenarios Tested | E.g. Adding 1 nurse, changing policy on alcohol taxation… |
| Key Simulation Output | E.g. waiting time, LOS… |
| Recommendations from simulation | E.g. should add another nurse before investing in more beds |

**Stakeholder Engagement details:**

| Approach/methodology for stakeholder engagement incl. terminology | E.g. "participatory modelling", "fully-facilitated DES"… |
| --- | --- |
| + details about methodology if given | - Existing, adapted, or novel? - Derived from which field? |
| Types of stakeholders involved incl. terminology | E.g. patients, nurses, senior consultants, "problem owners"… |
| When were stakeholder engaged? | E.g. during problem structuring, during implementation stage… |
| What activities did the stakeholders get involved with? | E.g. Providing investigational components/scenarios to test, contributing mental model of system to the conceptual model… |
| Modes of engagement | E.g. interview, survey, focus group … |
| Mechanisms of interaction | E.g. webinar, face-to-face … |
| Special provision for patients if involved? | E.g. extra information given about the topic, incentives provided… |
| Freq/duration of contact | E.g. 3 workshops one 4hrs each |
| Dissemination method | E.g. Delphi method, shared decision-making, qualitative synthesis, quantitative survey … |
| Influence of stakeholders on model purpose, design or structure | E.g. Since end-users have no influence on X part of the system, this was only modeled in a simplified way |
| Expected/intended positive outcomes of stakeholder engagement | - Quoted - Framework from Seidl, 2015 |
| Reported positive outcomes of stakeholder engagement | - Quoted - Framework from Seidl, 2015 |
| Reported negative outcomes of stakeholder engagement |  |
| Reported enablers of engagement of stakeholders |  |
| Reported barriers to engagement of stakeholders |  |
| Implementation Level | - Theoretical (proposed by authors) - Conceptualized (discussed with client organisation) - Implemented (changes attempted within organisation on the back of simulation study) |
| Success reported based on four stage model of success (Robinson & Pidd, 1998) | - Stage 1: The study achieves its objectives and/or shows a benefit; - Stage 2: The results of the study are accepted; - Stage 3: The results of the study are implemented; - Stage 4: Implementation proved the results of the study to be correct |
| Successful elements of implementation and enablers |  |
| Unsuccessful elements of implementation and barriers |  |
| Other lessons learned |  |
| Resources associated with stakeholder engagement activities |  |

**Data analysis and synthesis**

Data will be presented in summary form and descriptively, in tables or narratively for each clinical question.

**Discussion**

Optional - revisit the justification for the systematic review and clinical gap and discuss what this systematic review will add to the literature and its likely clinical impact.

- Participatory models more generally referring to consumer and community engagement for qualitative data collection but not linked to simulation – community-based participatory research (CBPR)
- Limitations in drawing the line for stakeholder engagement, specifically at the level of abstracts. Conclusion made that is insufficient information is given about engagement of stakeholders at the level of the abstract then the focus of the article is unlikely to be strong in this area
- Limitations in search strategy, difficult to filter out the in-situ, interprofessional simulations etc. as many of these studies were left.
- Many environmental participatory situation modelling articles coming up which mention health. These articles give much more detail about the participative aspect of the methodology than the health journals.
- Final conflicts between KL and TZ were articles which did not mention stakeholder involvement in the modelling process explicitly but had reference to stakeholders commissioning the model and being involved for scenario generation and at times implementation. These were resolved in line with the criteria that stakeholder engagement to influence model design must be mentioned in the title/abstract in order to meet inclusion criteria. This is a limitation of this systematic review and shows the need for clearer reporting around the engagement and involvement of stakeholder in helping to conceptualize the model and being included in decision-making about model design.
- Many articles implicitly seem to involve stakeholders but don’t explicitly state this*include insufficiently reported reason for excluding at full-text level
- Those that were excluded for being theoretical only could be analyzed for different things
- Only compared disagreements Yes/No and nothing to do with reasons
- Asked for second opinion on borderline articles
- System dynamics was the only type of modelling before certain time so they wouldn’t have stated this.
- Really difficult to make the distinction between expected benefits of engaging stakeholders in the simulation modelling and reported benefits that were actually observed or indicated during the process.

**Table 1. Selection criteria**

**Title & Abstract Screening**

|  | **Stakeholder Engagement** | **Simulation Modelling** | **Healthcare** |
| --- | --- | --- | --- |
| **Inclusion criteria** | Stakeholders engagement - abstract must refer to:  - Stakeholders are involved in the process before the stage of implementing the model.  - Engagement must occur before computer simulation model is built i.e. during development and building of the model | - computational simulation only  - system dynamics, discrete event simulation and/or agent-based modelling. If type not stated at abstract level, citations will be included. | - Project or case study focussing on a health care/mental health care issue - from epidemiology up to policy level simulation models.  - Simulation models which look at the impact of wider changes on health will be included as long as health/health care is the primary consideration of the model e.g. alcohol, drug misuse, smoking will be included if the impact of changes on health/health care system is primary aim. Disability-related studies are included. |
| **Exclusion criteria** | - Stakeholders are only engaged at the stage of implementation  - Provision of interventions/scenarios to test only  - Validation of model only, unless used to iteratively improve model  - "Expert opinion" used to parameterize the model  - Existing surveys used | - Markov models, montecarlo simulation etc. unless combined with SD, DES or ABM.  - Any non-computational type of simulation e.g. Clinical simulation, high-fidelity simulation, simulation training etc | - Primary aim of model other than health e.g. environmental air pollution, effect of drugs/alcohol on crime.  - Dentistry-related topics are excluded  - Simulation models of medical information storage only are excluded.  - Molecular-level simulations are excluded. |

**Full-text screening**

|  | **Stakeholder Engagement** | **Simulation Modelling** | **Healthcare** |
| --- | --- | --- | --- |
| **Inclusion criteria** | Minimum information in full-text to be included is:  1. who the stakeholders were (descriptions such as "problem owner" is sufficient)  2. when they were involved (at what stage of the project the stakeholders were involved)  3. what the function, expected benefit or actual benefit was of the engagement (could be process-driven e.g. "to inform stakeholders about results" or "facilitate social learning" or outcome-driven e.g. "increase acceptance of model") | - computational simulation only  - system dynamics, discrete event simulation and/or agent-based modelling. Must state this at the full-text stage. Hybrid simulation models using a least one of these types will be included - can be combined with another type of modelling. | - Project or case study focussing on a health care/mental health care issue - from epidemiology up to policy level simulation models.  - Simulation models which look at the impact of wider changes on health will be included as long as health/health care is the primary consideration of the model e.g. alcohol, drug misuse, smoking will be included if the impact of changes on health/health care system is primary aim. Disability-related studies are included. |
| **Exclusion criteria** | - Stakeholders are only engaged at the stage of implementation  - Provision of interventions/scenarios to test only  - Validation of model only, unless used to iteratively improve model  - "Expert opinion" used to parameterize the model  - Existing surveys used | - Markov models, montecarlo simulation etc. unless combined with SD, DES or ABM.  - Any non-computational type of simulation e.g. Clinical simulation, high-fidelity simulation, simulation training etc | - Primary aim of model other than health e.g. environmental air pollution, effect of drugs/alcohol on crime.  - Dentistry-related topics are excluded  - Simulation models of medical information storage only are excluded.  - Molecular-level simulations are excluded. |

**References**

Bramer, W. M., Rethlefsen, M. L., Kleijnen, J., & Franco, O. H. (2017). Optimal database combinations for literature searches in systematic reviews: A prospective exploratory study. *Systematic Reviews*, *6*(1), 245. <https://doi.org/10.1186/s13643-017-0644-y>

Chen, J. (2018). *Agent-based Modelling in Healthcare Operations: A Systematic Review of its Scope, Quality and Implementation* [University of Oxford]. <https://ora.ox.ac.uk/objects/uuid:9b71bc9f-61c1-4485-93b2-0b7e17e0e62f/download_file?file_format=pdf&safe_filename=SysReview%2Bof%2BABM%2Bin%2BHealthcare%2BOperations%2B%25281010130%253B%2B2017-2018%2BDissertation%2529.pdf&type_of_work=Thesis>

Concannon, T. W., Fuster, M., Saunders, T., Patel, K., Wong, J. B., Leslie, L. K., & Lau, J. (2014). A Systematic Review of Stakeholder Engagement in Comparative Effectiveness and Patient-Centered Outcomes Research. *Journal of General Internal Medicine*, *29*(12), 1692–1701. <https://doi.org/10.1007/s11606-014-2878-x>

Esmail, L., Moore, E., & Rein, A. (2015). Evaluating patient and stakeholder engagement in research: Moving from theory to practice. *Journal of Comparative Effectiveness Research*, *4*(2), 133–145. <https://doi.org/10.2217/cer.14.79>

Robinson, S., & Pidd, M. (1998). Provider and Customer Expectations of Successful Simulation Projects. *The Journal of the Operational Research Society*, *49*(3), 200–209. JSTOR. <https://doi.org/10.2307/3010469>

Workman, T., Maurer, M., & Carman, K. (2013). Unresolved tensions in consumer engagement in CER: A US research perspective. *Journal of Comparative Effectiveness Research*, *2*(2), 127–134. <https://doi.org/10.2217/cer.13.6>
